# Mapping cross-modal functional connectivity of major neurotransmitter systems in the human brain

**DOI:** 10.1007/s00429-025-02996-4

**Published:** 2025-08-19

**Authors:** C. Saiz-Masvidal, V. De la Peña-Arteaga, S. Bertolín, I. Diez, A. Juaneda-Seguí, I. Martínez-Zalacaín, P. Chavarría-Elizondo, M. Subirà, J. M. Menchón, J. Sepulcre, Miquel Àngel Fullana, Carles Soriano-Mas

**Affiliations:** 1 Psychiatry and Mental Health Group, Neuroscience Program, Bellvitge Biomedical Research Institute (IDIBELL), L’Hospitalet de Llobregat, Spain; 2 Department of Clinical Sciences, School of Medicine, University of Barcelona, L’Hospitalet de Llobregat, Spain; 3 Sant Pau Mental Health Research Group, Institut de Recerca Sant Pau (IR SANT PAU), Barcelona, Spain; 4 Department of Psychiatry, Bellvitge University Hospital, Hospitalet de Llobregat, Barcelona, Spain; 5 Network Center for Biomedical Research on Mental Health (CIBERSAM), Carlos III Health Institute (ISCIII), Madrid, Spain; 6 Gordon Center for Medical Imaging, Department of Radiology, Massachusetts General Hospital, Harvard Medical School, Boston, MA USA; 7 Athinoula A. Martinos Center for Biomedical Imaging, Department of Radiology, Massachusetts General Hospital, Harvard Medical School, Boston, MA USA; 8 Radiology Department, Hospital Universitari de Bellvitge, L’Hospitalet de Llobregat, Carrer de Feixa Llarga SN, Barcelona, 08907 Spain; 9 Department of Radiology, Yale PET Center, Yale Medical School, Yale University, New Haven, CT USA; 10 Institut d’Investigacions Biomèdiques August Pi i Sunyer, Barcelona, Spain; 11 Psychiatry and Psychology Service, Clinical Institute of Neurosciences, Hospital Clínic, Barcelona, Spain; 12 Department of Social Psychology and Quantitative Psychology, Institute of Neurosciences, University of Barcelona, Bellvitge Biomedical Research Institute (IDIBELL) and CIBERSAM, Barcelona, Spain

**Keywords:** Monoaminergic signaling, Resting-state functional magnetic resonance imaging, Resting-state functional connectivity, Resting-state networks, Neurotransmitter mapping

## Abstract

Monoaminergic systems, including serotonin, dopamine, and norepinephrine, are essential for regulating brain activity and facilitating behavioral flexibility. These systems originate from brainstem nuclei and project widely to modulate functions such as mood, attention, memory, and adaptability. Using resting-state functional MRI (rs-fMRI), this study aimed to investigate the connectivity networks of key monoaminergic nuclei in 193 healthy adults and explore their correspondence with molecular imaging maps of neurotransmitter-specific biochemical markers. Functional connectivity (FC) was assessed using seed-based rs-fMRI analyses with seeds placed in the dorsal raphe nucleus (DRN), nucleus centralis superior (NCS), ventral tegmental area (VTA), substantia nigra pars compacta (SNc), and locus coeruleus (LC). Cross-modal analyses using molecular imaging data were performed to correlate these rs-FC maps with the distribution of neurotransmitter-related receptors, transporters, and synthesis enzymes, providing insights into the molecular architecture underlying the FC of monoaminergic systems. Whole-brain FC maps revealed distinct patterns for each nucleus. DRN projections were extensive, connecting to subcortical regions such as the hippocampus and amygdala and cortical areas including the precuneus, cingulate, and medial frontal cortex. NCS projections overlapped partially but uniquely targeted the orbitofrontal and insular cortices. Dopaminergic pathways exhibited connectivity with the striatum, thalamus, and prefrontal cortex, while noradrenergic LC projections displayed lateralized connectivity to occipital, temporal, and frontal regions. Cross-modal correlations with molecular imaging demonstrated significant spatial associations between rs-FC maps and neurotransmitter-specific markers, including 5HTT, DAT, and FDOPA. This study enhances our understanding of neurotransmitter networks, highlighting their relevance in brain function and potential as biomarkers for neuropsychiatric conditions.

**Supplementary Information:**

The online version contains supplementary material available at 10.1007/s00429-025-02996-4.

## Introduction

Neuromodulatory systems serve as a critical mechanism for brain activity regulation, enabling flexible navigation through diverse behavioral states (Bär et al. 2016; O’Callaghan et al. 2021). These systems primarily arise from small-molecule neurotransmitters, especially monoamines, including the indolamine serotonin (5-hydroxytryptamine; 5-HT) and the catecholamines dopamine (DA) and norepinephrine (NE) (Bär et al. 2016; Gillespie et al. 2020; Shao And Zhu 2020). Monoaminergic neurons, strategically positioned within distinct nuclei across the brainstem, covering midbrain and hindbrain areas (Carandini et al. 2021; Izzi and Charron 2013), project long axons towards the forebrain. These projections significantly influence the functioning of hierarchical neural pathways (Gu 2002; Hensler et al. 2013), playing an essential role in managing a broad spectrum of physiological and psychological functions, including emotion, arousal, attention, memory, and various aspects of motivated behavior, such as reward processing, reinforcement learning, and behavioral flexibility (Carandini et al. 2021; Charnay and Léger 2022; Gu 2002; Peters et al. 2021). Thus, the correct balance of these systems contributes to optimal brain functioning, being their dysregulation and maladjustment a potential trigger for a wide spectrum of mental illnesses (Huang et al. 2019; Shao and Zhu 2020; McCarty et al. 2021; Wagner et al. 2017).

The serotonergic system, originating from the upper raphe nuclei—the dorsalis raphe nucleus (DRN) and nucleus centralis superior (NCS)— extensively innervates almost every brain region (Walker and Tadi 2023), and has been implicated in a wide variety of behavioral and neurological disorders (Huang et al. 2019; Kenna et al. 2012). These highly divergent projections target many functionally distinct brain regions modulating arousal, motor facilitation, behavioral, cognitive and social flexibility, mood regulation and memory (Huang et al. 2019; Walker And Tadi 2023). Similarly, the major dopaminergic pathways, such as the nigrostriatal pathway from the substantia nigra pars compacta (SNc) and the mesocorticolimbic pathway from the ventral tegmental area (VTA), regulate movement and motivated behaviors, and influence executive, affective, and motivational functions, respectively (Conio et al. 2020; Yang et al. 2020). Finally, the locus coeruleus (LC), situated in the rostral pontine brainstem, is the primary source of NE, extensively projecting throughout the cerebral cortex and various subcortical structures (Liu et al. 2017; Zhang et al. 2016; Pitzer 2019). This arrangement is pivotal for enabling rapid and global brain function modulation in response to environmental stimuli (Bremner et al. 1996; Keren et al. 2009; Pitzer 2019; Shao and Zhu 2020).

Due to their extensive and targeted projections to distantly located brain regions, these neurotransmitter systems can rapidly influence cortical network activity (Conio et al. 2020). Previous research indicates that the organization of monoamine neurotransmitter systems aligns with the complex interactions among large-scale neural networks regulating essential brain functions (Sporns 2022; Stramaglia and Cortes 2022; Tymofiyeva et al. 2014). These include executive function, motor coordination, and emotional regulation (Hensler et al. 2013). Such interactions manifest as statistical patterns of functional connectivity (FC) in resting state brain activity, suggesting that neurotransmitter-related neuronal activity may synchronize low-frequency oscillations across these networks, influencing their baseline activity and balance (Conio et al. 2020).

The analysis of resting state-functional connectivity (rsFC) from functional magnetic resonance imaging (fMRI) data allows for the assessment of the monoaminergic connectivity networks of these different neurotransmitter systems (Saiz-Masvidal et al. 2024). In addition, recent studies have highlighted the importance of integrating functional imaging techniques with molecular neurobiology to better understand the neurophysiological mechanisms underlying brain activity (Dipasquale et al. 2019; Dukart et al. 2021; Oldehinkel et al. 2022; Savio et al. 2017). Techniques such as positron emission tomography (PET) and single-photon emission computed tomography (SPECT) allow the derivation of tissue property maps, including the distribution of synthesis enzymes, reuptake processes, and receptors (Li et al. 2018; Van Spronsen and Hoogenraad 2010). When compared with rsFC data, these maps have revealed that spatial patterns in functional connectivity are often associated with the distribution of specific receptor systems (Dukart et al. 2021; Oldehinkel et al. 2022). This connection provides valuable insight into the biochemical underpinnings of brain circuits. Notably, these imaging studies suggest that neurotransmitter-related resting-state maps reflect the broader biochemical architecture of the brain, yet whether these functional patterns consistently correspond to the receptor systems of their respective neurotransmitters remains an open question (Dukart et al. 2021).

This study aims to investigate the connectivity network of monoamine neurotransmitter nuclei within the healthy human brain. Employing a seed-based methodology, we aim to pinpoint regions exhibiting significant rsFC with each nucleus. Furthermore, the study also aims to determine if the connectivity maps for each neurotransmitter correspond spatially with the distribution of their specific biochemical machinery, such as receptor systems, as can be mapped by nuclear medicine techniques (Dukart et al. 2021). By adopting this cross-modal strategy, we intend to augment our investigation of neurotransmitter-related networks with insights into the neurobiological mechanisms that facilitate interactions between distinct systems (Bosulu et al. 2022; Dukart et al. 2021). This approach seeks to validate the connectivity patterns observed through functional imaging techniques and to anchor these observations in the underlying molecular architecture of the brain, thereby providing a more comprehensive understanding of the complex interplay between neurochemical signaling and neural network dynamics.

## Methods

### Participants

A total of 193 healthy adults (100 females, mean age ± SD (range) = 25.7 ± 4.96 (18–46)) were assessed.

The inclusion/exclusion criteria for our study were defined as follows: participants must be aged 18 to 50, demonstrate a willingness and eligibility to undergo magnetic resonance imaging (MRI), and have no history or current diagnosis of severe medical or mental disorders. Additionally, individuals with current substance use were excluded, except for tobacco and the occasional use of alcohol or other recreational drugs. This was confirmed with WHO - ASSIST V3.0 interview. Those meeting these criteria were asked to provide written informed consent before participating in the study. This study received approval from the Ethics Committee for Clinical Research at Bellvitge University Hospital. It was conducted in strict adherence to the ethical standards outlined in the Declaration of Helsinki.

### Data acquisition

MRI data was acquired using a 3.0 Tesla MRI scanner (Ingenia, Philips Medical Systems, Eindhoven, Best, Netherlands) equipped with a 32-channel phased-array head coil. The functional MRI (fMRI) protocol for resting-state included 240 single-shot gradient-echo echo-planar imaging (EPI) volumes, captured with the following specifications: a repetition time (TR) of 2,000 ms, an echo time (TE) of 25 ms, and a flip angle of 90 degrees; within a 240-mm field of view (FOV); and an 80 × 80 matrix size, yielding voxel dimensions of 3 × 3 × 3 mm, with no inter-slice gap and 40 interleaved slices oriented parallel to the anterior-posterior commissure line for comprehensive brain volume coverage. The total duration of the resting-state sequence was 8 min. Additionally, a high-resolution T1-weighted anatomical scan was obtained to support the alignment of EPI data into the standard MNI space and for extracting the individual gray matter volume for each subject.

### Data preprocessing

The neuroimaging data underwent processing and analysis on a Microsoft Windows platform, utilizing MATLAB 9.3 (Release 2017b, The MathWorks, Inc.) and the CONN Functional Connectivity SPM Toolbox v20.b (www.nitrc.org/projects/conn). The preprocessing of functional images adhered to the Montreal Neurological Institute’s (MNI) default preprocessing guidelines within the CONN toolbox, encompassing realignment, unwrapping, and slice-timing correction. Structural volumes were segmented and normalized to MNI space, delineating gray and white matter as well as cerebrospinal fluid segments. This segmentation informed the preprocessing of functional data, which was subsequently smoothed using an 8 mm full-width at half maximum (FWHM) isotropic Gaussian kernel. Noise attributed to blood oxygenation level–dependent signals from white matter and cerebrospinal fluid was identified via the aCompCor method and removed in a subsequent denoising phase, which also included linear detrending to eliminate linear/quadratic/cubic trends within the functional session, and the application of a despiking process to mitigate the impact of potential outlier scans. In addition, to control for movement effects, individuals with movement artifacts were excluded in two steps: first, those with brain alterations identified through visual inspection, and second, those with more than 33% invalid scans due to movement. From an initial sample of 208 subjects, 15 subjects were discarded because of this reason, resulting in a final sample of 193 participants. Finally, to control for the confounding effects of cardiac and respiratory cycles, data were subjected to band-pass filtering within a frequency range of 0.008 to 0.09 Hz. Importantly, to ensure high image quality, the sequences underwent inspection for artifacts before and after each processing step.

### Seed definition

**Fig. 1:**
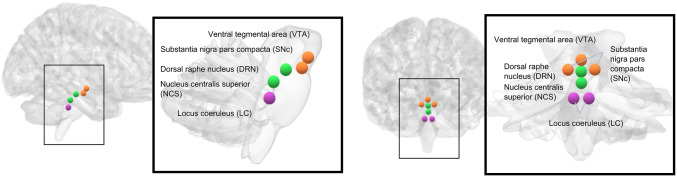
Sagittal (left) and coronal (right) three-dimensional rendering of the brainstem, highlighting the placement of neurotransmitter-related nuclei seeds. Those associated with the dopaminergic system are depicted in orange: the VTA is positioned at the central edge, with both SNc nodes situated laterally and slightly below the VTA. Seeds representing the serotonergic system are shown in green: the DRN) is located above and more ventrally, whereas the NCS is directly beneath it. Lastly, seeds in purple illustrate the noradrenergic system, corresponding to the bilateral LC

To assess the rsFC of neurotransmitter-related brainstem nuclei, we designated specific seeds (or regions of interest, ROIs) utilizing the MarsBar toolbox (available at http://marsbar-toolbox.github.io/marsbar). These ROIs were constituted as 3 mm radial spheres centered on selected bilateral MNI coordinates, which are graphically represented for clarity in Fig. 1.

For the serotonergic system, our analysis incorporated a seed within the dorsal raphe nucleus (DRN) at MNI coordinates [x = 0, y=−26, z=−18] and within the nucleus centralis superior (NCS) at [x = 0, y=−32, z=−24] (Sclocco et al. 2018). These locations house the majority of serotonergic neurons projecting throughout the brain, making them prime targets for a seed-based exploration of resting-state activity linked to 5-HT signaling (Bär et al. 2016; Beliveau et al. 2015). For the dopaminergic system, the VTA was designated at [x = 0, y=−15, z=−12] (Tomasi and Volkow 2014), and the bilateral SNc was identified at [x = ± 7, y=−18, z=−17] (Menke et al. 2010). The SNc coordinates were carefully selected to differentiate this region as distinctly as possible from the adjacent substantia nigra pars reticulata, based on the segmentation provided by Menke et al. (2010). Lastly, for the noradrenergic system, we located the LC at [x = ± 4, y=−34, z=−32] (Del Cerro et al. 2020). These nuclei are recognized as primary sites for norepinephrine release within the brain (Schwarz and Luo 2015).

To mitigate the potential for signal overlap between seeds resulting from spatial smoothing, we ensured that seeds within each hemisphere were spatially separated by a distance greater than 8 mm, equivalent to one full width at half maximum (FWHM). This separation was verified using the Euclidean distance formula $\:\sqrt{{\left(x1-x2\right)}^{2}+{\left(y1-y2\right)}^{2}+{\left(z1-z2\right)}^{2}}$. ROI files were standardized across all participants, employing normalized-space ROIs to facilitate a consistent analytical framework. From each ROI, we derived the average blood oxygen level-dependent (BOLD) signal time series from the within ROI voxels.

### Image analysis

As a general workflow, 1/. we initially utilized seed-based rs-fMRI techniques to delineate the connectivity patterns of the nuclei associated with the various monoaminergic systems, which allowed for the initial mapping and visualization of the connectivity networks for each neurotransmitter system. 2/. Subsequently, the final phase involved cross-modal analyses, wherein we investigated the spatial correlation between the rsFC maps and data derived from nuclear imaging techniques. This step aimed to uncover the biological underpinnings of the connectivity patterns observed.

Seed-based functional connectivity.

After data preprocessing, the data analysis was conducted using the CONN Functional Connectivity toolbox. In first-level analyses, seed-based correlation maps were generated to characterize the whole-brain rsFC for each of the seven seed ROIs at the subject-level. To achieve specificity in our findings, we employed semi-partial correlations for each system-related seed to mitigate the effects of nearby seeds. This approach was pivotal in isolating the signal attributable to the seed of interest. Nonetheless, to ensure that potential insights were not overlooked, we also conducted bivariate correlation analyses without adjusting for nearby system-related seeds (see Supplementary Figure S1).

These maps were then subjected to second-level general linear model (GLM) analyses within CONN to model data across subjects and delineate each ROI’s comprehensive whole-brain FC map. This analysis incorporated both positive and negative correlations, with significance determined at a statistical threshold of *p* < 0.05, employing cluster-level family-wise error (FWE) correction to account for multiple comparisons.

Cross-modal spatial correlations between fMRI data and nuclear imaging.

To explore the brain’s molecular architecture, we employed the JuSpace toolbox v1, a Matlab-based software designed for spatial correlation analysis between MRI data and neurotransmitter maps from PET and SPECT imaging (https://github.com/juryxy/JuSpace; Dukart et al. 2021). This tool was instrumental in correlating our seed-based rsFC maps with the spatial distribution of receptors within the serotonergic and dopaminergic systems. Additionally, we incorporated a nuclear imaging map related to norepinephrine (NE) neurotransmission (Hansen et al. 2022).

Our comprehensive cross-modal analysis included a suite of nuclear imaging maps targeting various receptors: five associated with serotonin (5HT_1a_, 5HT_1b_, 5HT_2a_, 5HT_4_, and the serotonin transporter 5HTT, or SERT); three related to dopamine (D_1_, D_2_ receptors, and the dopamine transporter DAT); and one each for DA synthesis (FDOPA), the mu-opioid receptor (MU, also termed MOP)—a key modulator of dopamine firing (Fields And Margolis 2015)—and the NE-specific presynaptic norepinephrine transporter (NET) (Schlessinger et al. 2011). For further information regarding the neurotransmitter and transporter maps, refer to Table 1.

**Table 1:** Neurotransmitter receptor/transporter maps included in the cross-modal Spatial correlation analyses

Receptor/transporter	Neurotransmitter	Tracer	*N*	Source/references
5-HT1a	Serotonin	[^11^C]WAY-100,635	36	https://neurovault.org/collections/1206/
5-HT1b	Serotonin	[^11^C]P943	22	Savli et al. 2012
5-HT2a	Serotonin	[^18^F]altanserin	19	
5-HT4	Serotonin	[^11^C]SB20	59	Beliveau et al. 2017
5HTT/SERT	Serotonin	[^11^C]DASB	30	
D1	Dopamine	[^11^C]SCH23390	13	Kaller et al. 2017
D2	Dopamine	[^18^F]fallypride	49	Jaworska et al. 2020
DAT	Dopamine	[^123^I]-FP-CIT	174	Dukart et al. 2018
FDOPA	Dopamine	[^18^F]fluorodopa	12	https://www.nitrc.org/projects/spmtemplate Gómez et al. 2018
MU/MOP	Opioid	[^11^C]Carfentanil	n/a	
NET	Norepinephrine	[^11^C]MRB	77	Hansen et al. 2022

*5-HT* 5-hydroxytryptamine; *D* dopamine; *DAT* dopamine transporter; *FDOPA* fluorodopa; *MU/MOP* mu-opioid peptide; *N* number of subjects used for map construction; *NET* norepinephrine transporter; *SERT (or 5HTT)* serotonin transporter

The cross-modal analysis workflow implemented in the JuSpace Toolbox followed these steps:

Data Preparation:

- *MRI data*: We incorporated the rsFC t-maps from our previous seed-based analyses limited to a single modality (using “files 1” only).
- *PET data*: We selected PET images that represent specific neurotransmitter receptor distributions. All PET maps were derived from average group maps of different healthy volunteers and linearly rescaled to range from a minimum of 0 to a maximum of 100, as per Dukart et al. 2018.

Preprocessing:

- *Spatial normalization*: Both MRI and PET images were aligned to a standard anatomical space to ensure accurate comparison.
- Parcellation: Brain images were segmented into regions of interest (ROIs) using predefined atlases. This approach allowed us to extract mean regional values from the MRI data for correlation with values from the PET and SPECT maps. We used a default atlas with 119 regions to provide a conservative estimate of the effective degrees of freedom, reducing potential for overly inflated degrees (Dukart et al. 2018).

Analysis:

- *Correlation computation*: We conducted Pearson partial correlation analyses to explore spatial relationships between our initial seed-based FC t-maps and PET data. Adjustments were made for spatial autocorrelation using the gray matter probability map TPM.nii from SPM12. Specifically, we chose the computing option that extracts the mean value per atlas region for each file.
- *Statistical testing*: Correlation outputs and significance values were calculated. We applied a stringent correction for multiple comparisons by setting the significance threshold at an FDR *p* < 0.05.

## Results

### Mapping of whole-brain RsFC from the different brainstem nuclei

#### Serotonergic system

**Fig. 2:**
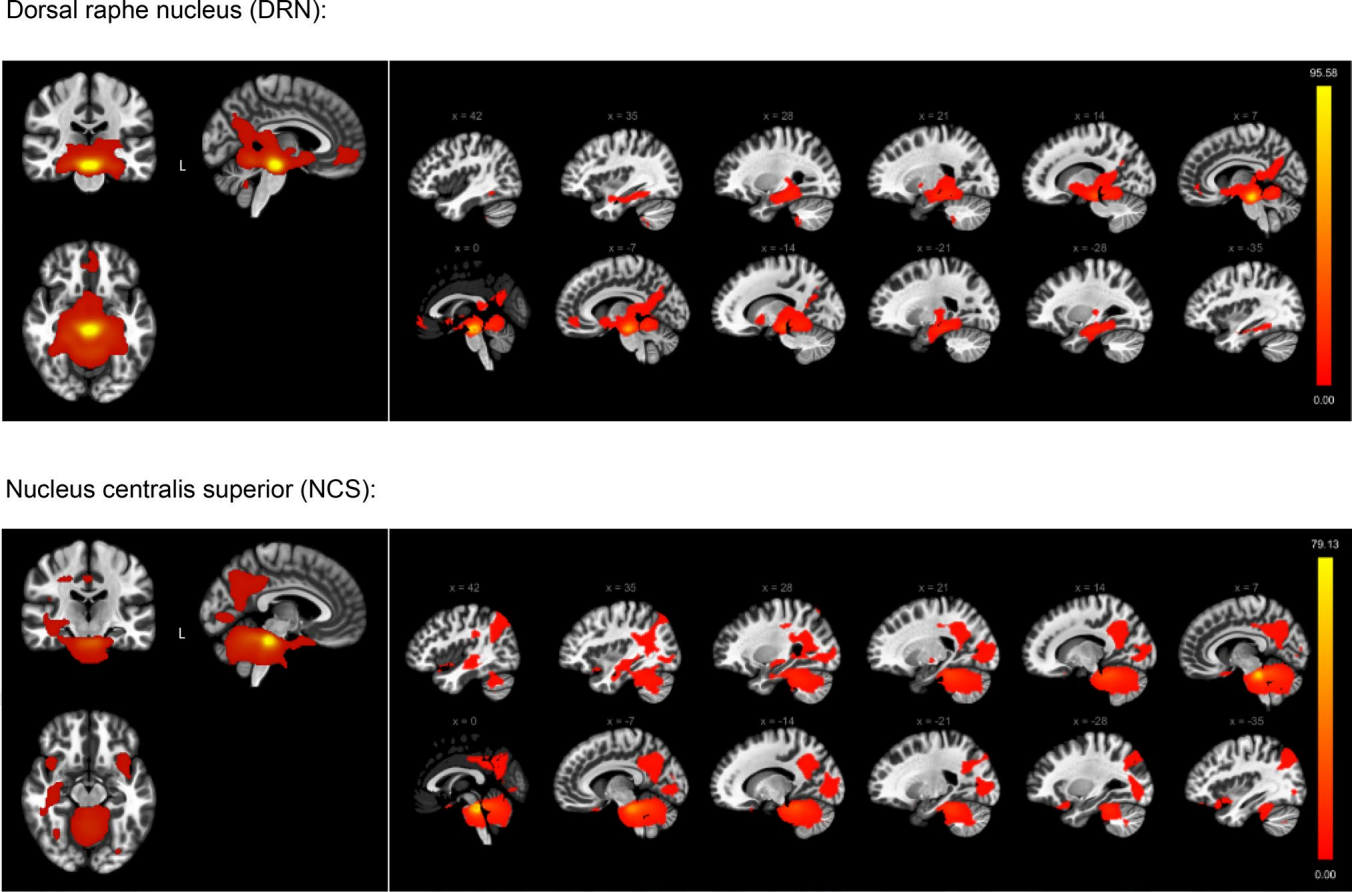
Functional connectivity projections originating from the dorsal raphe nucleus (DRN) (top) and from the nucleus centralis superior (NCS) (bottom), overlaid onto a normalized anatomical template. The images display the results of positive connectivity for each seed, with significance thresholds set at *p* < 0.05, adjusted for multiple comparisons using cluster-level family-wise error (FWE) correction. The color bar to the right displays the values of the t-test statistics

The serotonergic system, specifically from the DRN, exhibited extensive projections across a broad spectrum of subcortical areas, encompassing the brainstem, thalamus, hippocampal regions, and the amygdala. The projections also extended to various cortical regions, including the precuneus, posterior cingulate gyrus, lingual gyrus, and fusiform cortex. Furthermore, the DRN’s influence reached anteriorly to include the medial frontal cortex and paracingulate gyrus. Remarkably, the cerebellum and vermis were also among the recipient regions of DRN projections, as illustrated in Fig. 2.

In parallel, projections from the NCS displayed a degree of overlap with DRN projections, notably targeting the brainstem and hippocampal regions. Cortically, the NCS projections were observed in the precuneus cortex, posterior cingulate cortex, angular gyrus, and temporal occipital fusiform cortex, along with the lateral occipital cortex and occipital pole. Additionally, there was a bilateral extension of these projections to the frontal orbital cortex and insular cortex. A noteworthy feature of the NCS projections was their extensive reach to a multitude of cerebellar regions, further detailed in Figure 2.

#### Dopaminergic system

**Fig. 3:**
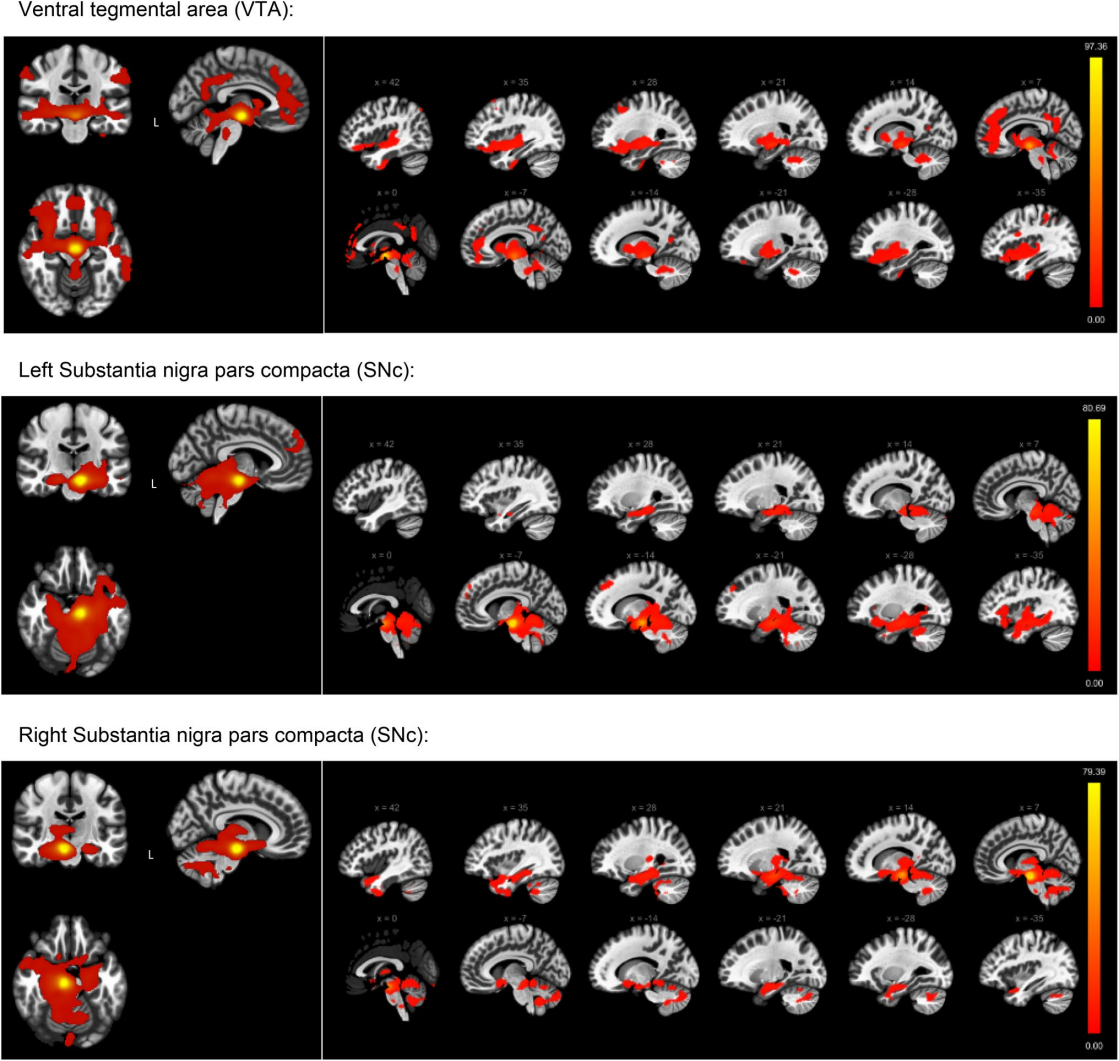
Functional connectivity projections originating from the ventral tegmental area (VTA) (top), the left substantia nigra pars compacta (SNc) (middle), and the right SNc (bottom), overlaid onto a normalized anatomical template. The images display the results of positive connectivity for each seed, with significance thresholds set at *p* < 0.05, adjusted for multiple comparisons using cluster-level family-wise error (FWE) correction. The color bar to the right displays the values of the t-test statistics

The dopaminergic projections from the VTA were observed to extend to both cortical and subcortical regions. Within the subcortical domain, target areas included the thalamus, brainstem, striatal regions (notably the putamen and pallidum), amygdala, and the nucleus accumbens, with the latter region receiving projections encompassing less than 100 voxels. Cortically, the VTA predominantly projected to the insula, frontal and temporal lobes, cingulate and paracingulate gyrus, and parietal regions, including the precuneus. Additionally, the cerebellum received a significant number of projections from the VTA, as depicted in Fig. 3.

Projections from the left SNc primarily targeted subcortical areas, such as the brainstem, hippocampus, thalamus, and amygdala. Moreover, these projections extended to the left frontal and temporal regions, and broadly to the bilateral cerebellum. The right dopaminergic SNc demonstrated projections to contralateral areas analogous to those targeted by the left SNc, with additional projections to certain subcortical regions, including the nucleus accumbens (also receiving less than 100 voxels), as illustrated in Fig. 3.

#### Noradrenergic system

**Fig. 4:**
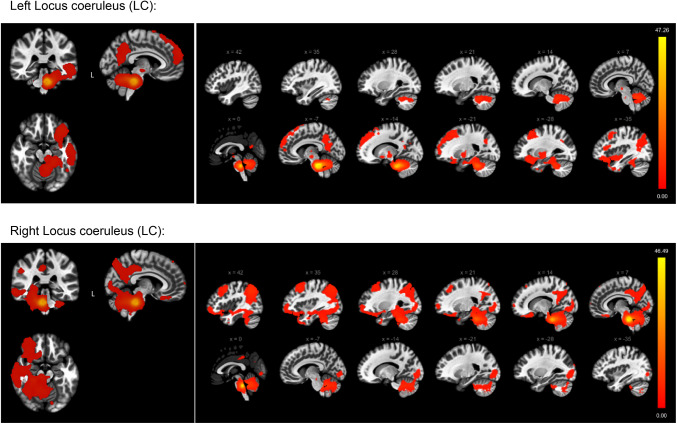
Functional connectivity projections originating from the left Locus Coeruleus (LC) (top), and the right LC (bottom), overlaid onto a normalized anatomical template. The images display the results of positive connectivity for each seed, with significance thresholds set at *p* < 0.05, adjusted for multiple comparisons using cluster-level family-wise error (FWE) correction. The color bar to the right displays the values of the t-test statistics

The noradrenergic projections emanating from the LC exhibited a pronounced lateralization towards the hemisphere of origin. Across both hemispheres, our analysis revealed that the predominant targets of these projections encompassed the occipital and frontal cortices, precuneus, angular gyrus, and temporal regions. Notably, the cerebellum emerged as a significant destination for LC projections, highlighting its importance within the noradrenergic network. Furthermore, the brainstem, along with various minor subcortical regions, also received noradrenaline-related projections, as meticulously documented in Fig. 4.

The results involving > 100-voxel clusters from these semipartial correlation analyses, which accounted for the influence of neighboring seed regions, are presented in Supplementary Table S1. The results of the bivariate correlation analyses, which do not consider the effects of adjacent seed regions, are displayed in Supplementary Figure S2.

Finally, to enhance visualization and simplify comparisons between the serotonergic and dopaminergic systems, individual maps of each serotonergic and dopaminergic seed are compiled in Supplementary Figure S2. This arrangement allows for direct comparison of the connectivity maps of these two monoaminergic systems. Additionally, a comprehensive serotonergic global map that combines the FC of the DRN and NCS seeds, a dopaminergic global map integrating the FC of the VTA, left SNc, and right SNc seeds, and a norepinephrine global map that consolidates the FC of both LC seeds are presented in Supplementary Figure S3 to facilitate comparisons across systems.

### Cross-modal correlations between RsFC and nuclear imaging neurotransmitter maps

**Fig. 5:**
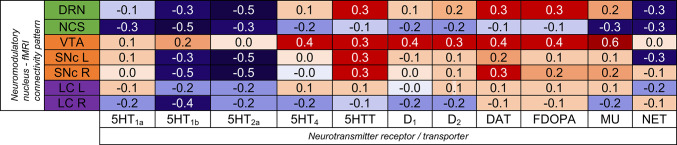
Correlation matrix heatmap showing FDR-significant positive correlations between rsFC maps (y-axis: neuromodulatory nucleus - fMRI connectivity pattern) and nuclear imaging maps (x-axis: neurotransmitter receptor/transporter)

Significant positive spatial correlations between rsFC maps and nuclear imaging maps of neurotransmitter-related compounds are summarized in Fig. 5, Supplementary Figure S4, and Supplementary Table S2. Specifically, for the DRN FC map, correlations were observed with the distribution of 5HTT, DAT and FDOPA. The VTA FC map exhibited significant positive correlations with the distribution of a broad spectrum of receptors including 5HT_4_, 5HTT, D_1_, D_2_, DAT, FDOPA, and MU.

For the left SNc FC map, correlations were specifically significant with the distribution of 5HTT. The right SNc FC map also showed significant correlations with 5HTT and DAT distribution maps. No significant correlations were identified for the NCS and the bilateral LC. However, in some of these correlations, Spearman’s values were noticeably lower than those of Pearson’s. This may indicate that these correlations could be influenced by the presence of outliers (see Supplementary Table S2 and the different panels Supplementary Figure S4). As a result, these correlations should be interpreted with caution.

## Discussion

In this study we explored the rsFC of the brain’s major monoaminergic modulatory systems. We linked these systems to a molecular level, shedding light on their biological basis observed through nuclear imaging of monoaminergic compounds. Our findings reveal unique and overlapping whole-brain functional connectivity patterns for the monoaminergic neurotransmitter nuclei, demonstrating their extensive influence across various brain structures.

The serotonergic system’s DRN and NCS influence both overlapping and distinct brain regions. They connect broadly from the brainstem to subcortical and cortical areas like the hippocampus and precuneus. Specifically, the DRN also reaches the thalamus, amygdala, and regions like the lingual gyrus and medial frontal cortex, whereas the NCS exclusively targets the insula and orbitofrontal cortex. Despite these differences, their overall connectivity patterns are relatively similar. This is supported by previous studies showing shared functional connectivity maps for these nuclei with major brain regions such as the cingulate and amygdala, demonstrating significant positive connectivity (Lorens et al. 1971; Bär et al. 2016; Beliveau et al. 2015; Charnay and Léger 2022; Hornung 2003).

The VTA demonstrates widespread neural connections across the brain, influencing regions including the brainstem, thalamus, hippocampus, and various forebrain and cortical areas such as the insula, consistent with Hansen et al. 2024. This extensive connectivity is supported by findings from Tomasi and Volkow (2012), who noted VTA’s links to the thalamus and hippocampus. Additional research by Bär et al. 2016; Gu et al. 2010; Hadley et al. 2014; Huang et al. 2021; and Murty et al. 2014 further corroborates the VTA’s connectivity with the brainstem, putamen, and cortical regions, reinforcing our observations. However, in our study, connectivity with the nucleus accumbens was less pronounced compared to its distinct target region status in other studies (Bär et al. 2016; Gu et al. 2010). For the SNc, both sides displayed lateral extensions with similar connections to the brainstem, thalamus, and cortical areas. The left SNc notably connects to the ipsilateral frontal pole, aligning with Hansen et al. 2024; which highlighted the association of this connectivity with autobiographical memory and social cognition; while the right SNc shows modest connections to the nucleus accumbens. These patterns are consistent with previous findings of SNc functional projections, although some areas identified in other studies were not observed here (Haber and Fudge 1997; Martino et al. 2018; Tomasi and Volkow 2014; Murty et al. 2014). Additionally, the presence of the 5HTT transporter in areas such as the precuneus, the cingulum, and the precentral and lingual gyrus, as documented in our DRN, VTA, and SNc FC maps, coincides with the findings reported by Dipasquale et al. 2019.

Finally, the LC’s noradrenergic projections are markedly lateralized, affecting a wide range of regions including the brainstem, hippocampal, and extensive prefrontal areas. The left LC also connects to the striatum. This matches with earlier research documenting positive LC connectivity with several cortical and subcortical areas, including the hippocampus and cerebellum (Huang et al. 2021; Kline et al. 2016; Liebe et al. 2022; Pitzer 2019; Zhang et al. 2016).

Notably, all monoaminergic nuclei displayed robust connectivity with the brainstem, thalamus, hippocampus, and cerebellum, showing shared connections that modulate brain regional activity (Bär et al. 2016; Zhang et al. 2016). Research suggests these neurotransmitter systems work together, influencing each other and their target circuits, leading to a network of overlapping connections that allows for coordinated neuronal activity (Briand et al. 2007; Hensler et al. 2013). Additionally, we found the prefrontal cortex (PFC) receives serotonin, dopamine, and norepinephrine projections from all nuclei, each targeting different PFC subregions. This underlines the unique roles of monoaminergic systems in affecting distinct PFC areas involved in cognitive and executive functions. Chandler et al. (2013) also suggested that DRN, VTA, and LC neurons could independently modulate specific PFC subregions, supporting our findings of their differentiated functional contributions.

In terms of monoaminergic neurotransmission within the constituent brain regions of large-scale networks, our findings reveal significant innervation by monoaminergic nuclei. Specifically, the DRN exhibited major connectivity with DMN areas such as the precuneus, posterior cingulate, and prefrontal medial cortex, highlighting the role of serotonin in introspection and social cognition (Buckner et al. 2008; Schrantee et al. 2018; Seitzman et al. 2019). This is consistent with multiple studies linking the serotonergic system to the DMN (Bär et al. 2016; Conio et al. 2020; Schrantee et al. 2018; Seitzman et al. 2019) while others have primarily found a DRN – executive control network (ECN) association (Janet et al. 2024). The NCS demonstrated functional associations with diverse RSNs, such as the DMN and the salience network (SN), suggesting serotonin’s role in maintaining vigilance and responding to salient stimuli (Seitzman et al. 2019). The VTA showed functional pathways innervating regions overlapping with the SN and sensorimotor network, aligning with findings by Conio et al. 2020; who reported functional connections with core areas of the SN. In the case of the SNc, it was found to innervate several areas across different RSNs, particularly the limbic cortex, which includes the orbitofrontal cortex, amygdala, hippocampus, and thalamus. These results underscore dopamine’s involvement in processes related to vigilance, arousal, emotion, motivation, memory, and affective experience (Phan et al. 2002; Seitzman et al. 2019). Connectivity pathways from the LC also overlapped with the visuospatial network, DMN, SN and the ECN, partially consistent with Bär et al. 2016; who noted the functional integration of the noradrenergic LC with the ECN and its involvement in goal-oriented behaviors and cognitive flexibility (Suttkus et al. 2021), and also within the SN playing a critical role in emotional processing (Geng et al. 2024). These insights into neurotransmitter systems emphasize their crucial role in the functional relationships within large-scale networks and their potential impact on network integration and modulation, associated with higher cognitive functions (McCarty et al. 2021).

In exploring the distribution of specific molecules linked to the functional projections of monoaminergic nuclei, we discovered both self and external contributions of neurotransmitter-related biochemical elements within the connectivity of different neurotransmitter systems. This modulation involves not only the molecular receptors unique to each system but also those belonging to other neurotransmitter systems. Specifically, the connectivity map related to the DRN was associated with the serotonin transporter (5HTT) as well as the dopaminergic transporter. The interaction between serotonin and other neural elements is primarily determined by serotonin’s extracellular concentration and the variety of its high-affinity receptors (Charnay and Leger 2022). Additionally, the presence of dopamine, norepinephrine, and other neuromodulators within serotonin neurons plays a role in serotonergic functions (Charnay a Leger 2022; Michelsen et al. 2008), with notable links between regional 5-HTT binding and DRN connectivity (Beliveau et al. 2015; Hansen et al. 2024). Similarly, maps related to dopaminergic nuclei were significantly correlated with both serotonergic and dopaminergic receptors, highlighting the importance of the interactions between serotonin and dopamine systems (Hansen et al. 2024; Vaseghi et al. 2022). Specifically, SNc connectivity showed an association with serotonergic (5HTT) and dopaminergic (DAT) transporters, which is in line with recent findings in which the FC network of a brainstem community that included the SNc was associated with these monoaminergic transporters (Hansen et al. 2024). Likewise, and also aligned with the findings of Hansen et al. 2024; the VTA was also associated to the MU opioid receptor.

In the literature, 5HTT is predominantly found in limbic areas like the thalamus, hypothalamus, amygdala, hippocampus, and striatum, with significant but lesser presence in cortical regions such as the frontal, temporal, parietal, and occipital lobes (Kish et al. 2005; Ressler And Nemeroff 2000). This distribution aligns with the locations shown in serotonergic and dopaminergic connectivity maps. Similarly, in agreement with our results, the highest concentrations of dopaminergic transporter are observed in the striatum and to a lesser degree in the amygdala, hypothalamus, hippocampus, some thalamic nuclei, and the neocortex (Piccini 2003). MU receptors are found in the somatosensory and limbic systems, matching the VTA projections to regions like the postcentral gyrus and limbic areas such as the hippocampus and amygdala (Herman & Muzio 2024; (Raju and Tadi 2022). Furthermore, consistent with other studies, the VTA connectivity pathway showed high affinity for several dopaminergic receptors such as 5HT_4_ D_1_ and D_2_, and FDOPA. The density of 5HT_4_ is relatively high in the caudate, putamen, accumbens and hippocampal formation, and lower elsewhere (Beliveau et al. 2017). This corresponds with the rs-FC map targets of the VTA, which uniquely involves the putamen and caudate (Supplementary Table S1). Regarding D_2_ receptor expression, it is highest in the striatal putamen and caudate and in thalamic regions, and more moderate but also significantly present in limbic and cortical temporal, parietal and occipital regions (Mukherjee et al. 2002). On the other hand, Kaller et al. 2017 found high density of D_1_ in the striatum and brain areas with low to moderate D_1_ expression such as the prefrontal cortex, consistent with our findings. Finally, also in line with our results, FDOPA biodistribution has been shown in the striatum (Chondrogiannis et al. 2013).

Our findings therefore emphasize the complex interplay between monoaminergic brain circuits and other neurotransmitter systems across multiple molecular dimensions (Charnay and Leger 2022). It has been suggested that monoaminergic transporters provide critical complementary insights into the FC cortical weighted degree, enhancing predictions across neurotransmitter systems (Hansen et al. 2024). Given the widespread projections of neurons in these nuclei, they potentially interact with nearly all other neuronal systems through a variety of heteroreceptors, creating a dense network of interactions among various neurotransmitter-related elements (Charnay and Léger 2022; Hahn et al. 2012). These interactions underscore the pivotal role of multiple neurotransmitter systems in modulating brainstem FC (Hansen et al. 2024).

rs-fMRI holds promise for understanding the implications of neurochemical changes in the brain, particularly in identifying the effects of typical and atypical neurotransmitter signaling related to various conditions (McCarty et al. 2021). This technology could significantly advance the detection of biomarkers for neurotransmitter-driven pathways, enhancing early disease detection and treatment monitoring (Hansen et al. 2024). For instance, rs-fMRI can detect changes in monoaminergic levels, offering insights into psychiatric disorders such as major depressive disorder, bipolar disorder, schizophrenia and obsessive-compulsive disorder (Huang et al. 2019; Shao And Zhu 2020; McCarty et al. 2021). This may generate more accurate models of disease propagation and aberrant dynamics, enabling the identification of precise brainstem targets for treatment interventions target (Hansen et al. 2024). Additionally, the ability of rs-fMRI to non-invasively monitor neurotransmitter network functions could refine therapeutic agent selection for neurological and neurodevelopmental disorders (McCarty et al. 2021). However, integrating information on neurochemical mechanisms with whole-brain hemodynamic responses remains a challenge, limiting explorations of pharmacokinetic and pharmacodynamic links (Dipasquale et al. 2019). In any case, the emerging field of pharmacological fMRI (pharma-fMRI) exemplifies the expanding applications of rs-fMRI underscoring the need to better understand how drugs influence brain activity and the BOLD signal (Anand et al. 2019; Weinstein et al. 2016). This advancement indicates the growing impact of rs-fMRI on monoaminergic research in the living human brain, offering a window into the complex interplay of neurochemistry and brain function (Bruinsma et al. 2018; Charnay and Léger 2022).

This study faces several challenges worth noting. Firstly, although substance use was controlled using the WHO - ASSIST V3.0 interview, we lacked specific data on substance consumption in the days immediately preceding the assessments. This includes substances like psilocybin that could potentially alter brain connectivity patterns. Nonetheless, participants were instructed to abstain from these substances before their fMRI scanning sessions. At the methodological level, we acknowledge that while the local gray matter probability adjustment implemented in the JuSpace toolbox helps mitigate inflated significance due to spatial clustering—by statistically controlling for local tissue probability—it does not fully address spatial autocorrelation. In particular, it does not account for spatial proximity or distance-based dependencies, which may result in an increased risk of false positives (Markello And Misic 2021). Another issue worth noting is that specific brainstem nuclei have distinct functional pathways and belong to separate circuits, making it difficult to study them in detail (Fields And Margolis 2015). The resolution of standard fMRI often falls short when identifying small areas like the midbrain in group images. Additionally, although our understanding of neurotransmitter systems has improved, and rs-fMRI has proven sensitive to detecting monoaminergic projections across the brain, there’s a pressing need for future research to enhance the sensitivity and specificity of rs-fMRI biomarkers related to neurotransmitters (McCarty et al. 2021). Enhancing the specificity and resolution of imaging techniques, with or without pharmacological interventions, will further our understanding of monoaminergic circuits in the human brain (Charnay and Léger 2022; Ganesana et al. 2017). In this context, employing high-field scanners and specialized brainstem-specific preprocessing pipelines could significantly enhance our precision in defining monoaminergic circuits. Nevertheless, our findings align well with recent studies that have utilized similar methodologies (Cauzzo et al. 2022; Singh et al. 2022). Looking ahead, integrating different imaging technologies, such as fMRI and PET, into hybrid-scanner systems represents a promising direction for advancing functional neuroimaging (Charnay and Léger 2022). Due to the high sensitivity and biochemical specificity of radiotracers, the use of simultaneous hybrid acquisition imaging may enrich our ability to investigate the neural underpinnings of rsFC and promote new insights into the physiological and molecular fingerprints underlying high-level neuronal organization, further helping to elucidate the basis of neuropsychiatric disorders (Aiello et al. 2016; Riedl et al. 2014).

## Conclusions

Monoaminergic nuclei extensively connect to both subcortical and cortical areas, impacting specific brain regions and indicating that neurotransmitter activity influences brain functional activity at many levels. This study reveals the intricate biological distribution of neurotransmitter system receptors across the brain, suggesting a complexity extending beyond simple pathways due to the widespread presence of heteroreceptors facilitating interactions across various systems. These insights mark significant progress in understanding monoaminergic rsFC, shedding light on its importance in brain function and its influence on emotional, cognitive, and behavioral outcomes. Such understanding underscores the potential of monoaminergic signaling as a biomarker for identifying dysfunction in neuropsychiatric conditions, enhancing our grasp of its broader role in brain dynamics (Beliveau et al. 2015; Tomasi and Volkow 2012).

## Supplementary Information

Below is the link to the electronic supplementary material.

Supplementary Material 1

## Data Availability

The data that support the findings of this study are available from the corresponding author upon reasonable request. Data are located in controlled access data storage at Bellvitge University Hospital.
