# Supplementary material for: Mapping cross-modal functional connectivity of major neurotransmitter systems in the human brain
Source: Brain Struct Funct. 2025 Aug 19;230(7):137. doi: 10.1007/s00429-025-02996-4 (PMC12364969; doi:10.1007/s00429-025-02996-4)
Supplement: Supplementary file 1 — Supplementary Material 1 [file 429_2025_2996_MOESM1_ESM.docx]

**Mapping cross-modal functional connectivity of major neurotransmitter systems in the human brain**

C. Saiz-Masvidal^1,2^, V. De la Peña-Arteaga^1,3^, S. Bertolín, M^1,2,4,5^., Diez, I^6,7^, A. Juaneda-Seguí^1,2^, I. Martínez-Zalacaín^1,8^, P. Chavarría-Elizondo^1,2,5^, M. Subirà ^4,5,^, J.M. Menchón^1,2,4,5^, Sepulcre, J^6,7,9^, M.A. Fullana^5,10,11^ C. Soriano-Mas^12^

^1^ Psychiatry and Mental Health Group, Neuroscience Program, *Bellvitge Biomedical Research Institute (IDIBELL)*, L’Hospitalet de Llobregat, Spain

^2^ Department of Clinical Sciences, School of Medicine, *University of Barcelona*, L’Hospitalet de Llobregat, Spain

^3^ Sant Pau Mental Health Research Group, Institut de Recerca Sant Pau (IR SANT PAU), Barcelona, Spain.

^4^ Department of Psychiatry, Bellvitge University Hospital, Hospitalet de Llobregat, Barcelona, Spain.

^5^ *Network Center for Biomedical Research on Mental Health (CIBERSAM), Carlos III Health Institute (ISCIII)*, Madrid, Spain

^6^ Gordon Center for Medical Imaging, Department of Radiology, Massachusetts General Hospital, Harvard Medical School, Boston, MA, USA

^7^ Athinoula A. Martinos Center for Biomedical Imaging, Department of Radiology, Massachusetts General Hospital, Harvard Medical School, Boston, MA, USA

^8^ Radiology Department, Hospital Universitari de Bellvitge, L'Hospitalet de Llobregat, Carrer de Feixa Llarga SN, 08907, Barcelona, Spain

^9^ Department of Radiology, Yale PET Center, Yale Medical School, Yale University, New Haven, CT, USA

^10^ Institut d’Investigacions Biomèdiques August Pi i Sunyer, Barcelona, Spain

^11^ Hospital Clinic, Barcelona, Spain

^12^ Department of Social Psychology and Quantitative Psychology, Institute of Neurosciences, University of Barcelona, and Bellvitge Biomedical Research Institute (IDIBELL) and CIBERSAM, Barcelona, Spain

*Corresponding Authors:

Carles Soriano-Mas, PhD

Department of Social and Quantitative Psychology

[Faculty of Psychology](https://www.google.es/maps/place/UB+Facultat+de+Psicologia/@41.4381216,2.1404858,17z/data=!3m1!4b1!4m5!3m4!1s0x12a497e1a4b38255:0x84c82132084ddaf0!8m2!3d41.4381216!4d2.1426745), University of Barcelona

Edifici de Ponent, Vall d’Hebron, 171

08035 Barcelona, Spain

E-mail: [carles.soriano.mas@ub.edu](mailto:carles.soriano.mas@ub.edu)

Telephone: +34 933125083

Miquel Àngel Fullana, PhD

Psychiatry and Psychology Service

Clinical Institute of Neurosciences, Hospital Clínic

C/ Villarroel, 170

08036 Barcelona, Spain

E-mail: [mafullana@clinic.cat](mailto:mafullana@clinic.cat)

Telephone: +34 932275477

**Supplementary material**

**Table S1**. Detailed results of brain regions comprised in significant clusters (*p*FWE<0.05) of seed-based functional connectivity analyses, corresponding to the findings reported in Figures 2 to 4.

| **Table S1.** Brain clusters showing significant positive functional connectivity with neurotransmitter nuclei | | | | | | | |
| --- | --- | --- | --- | --- | --- | --- | --- |
| seed | MNI coordinates | | | *k* | *p*_FWE-corr_ | Anatomic locations (>100vx) | |
|  | *x* | *y* | *z* |  |  |  |  |
| *Serotonergic system* | | | | | | | |
| **DRN** | +04 | -24 | -16 | 12912 | 0.000 | - Precuneus  - Brainstem  - Thalamus  - Hippocampus  - Cerebellum 3 4 5  - Vermis 3 4 5  - Cingulate G, post. div. | - Lingual G  - Parahippocampal G, post. div.  - Temporal Occipital Fusiform C  - Temporal Fusiform C  - Amygdala |
|  | -06 | +48 | -06 | 489 | 0.000 | - Frontal Medial C  - Paracingulate G | |
|  | +24 | -42 | -48 | 214 | 0.023 | - Cerebellum 8 R |  |
| **NCS** | +02 | -34 | -26 | 28836 | 0.000 | - Brainstem  - Precuneus  - Lateral Occ. C, sup. div.  - Hippocampus R  - Cerebellum 4 5 6 7b 8 9 Crus 1 2  - Intracalcarine C  - Vermis 1 2 3 4 5 6 8 9 10 | - Cingulate G, post. div.  - Temporal Fusiform C, post. div.  - Parahippocampal G, post. div.  - Occipital Pole  - Angular G |
|  | -36 | +14 | -16 | 628 | 0.000 | - Frontal Orbital C L  - Insular C L | |
|  | +38 | +14 | -16 | 201 | 0.036 | - Insular C R | |
| *Dopaminergic system* | | | | | | | |
| **VTA** | -02 | -12 | -10 | 18045 | 0.000 | - Insular C  - Frontal Orb. C  - Thalamus  - Middle Temporal G  - Brainstem  - Angular G  - Putamen  - Lateral Occ. C | - Pallidum  - Vermis 3 4 5  - Inferior Temporal G  - Hippocampus R  - Frontal Pole R  - Caudate  - Amygdala L |
|  | +02 | +40 | +34 | 3112 | 0.000 | - Paracingulate G  - Cingulate G, ant. div. | - Frontal Medial C  - Superior Frontal G |
|  | -50 | -28 | +38 | 1493 | 0.000 | - Supramarginal G L  - Postcentral G L | - Superior Parietal Lobule L |
|  | 00 | -58 | +14 | 1118 | 0.000 | - Precuneus C  - Cingulate G, post. div. | |
|  | +40 | -12 | -40 | 720 | 0.000 | - Temporal Fusiform C R  - Inferior Temporal G | |
|  | +64 | -26 | +44 | 423 | 0.001 | - Supramarginal G, ant. div. R  - Postcentral G R | |
|  | -42 | +02 | +22 | 298 | 0.005 | - Precentral G L | |
|  | +26 | +20 | +44 | 271 | 0.008 | - Middle Frontal G R | |
| **SNc L** | -10 | -20 | -16 | 15181 | 0.000 | - Brainstem  - Cerebellum 3 4 5 6 8 9  - Hippocampus  - Frontal Orbital C  - Vermis 3 4 5 6 9  - Temporal Pole  - Thalamus  - Insular C L  - Parahippocampal G | - Amygdala  - Lingual G  - Temporal Fusiform C L  - Frontal Operc. C L  - Middle Temporal G, ant. L  - Inferior Frontal G L  - Temporal Occipital Fusiform C L |
|  | -18 | +42 | +38 | 507 | 0.000 | - Frontal Pole L  - Superior Frontal G L | |
| **SNc R** | +06 | -18 | -20 | 13744 | 0.000 | - Brainstem  - Temporal Pole  - Thalamus  - Hippocampus  - Cerebellum 4 5 6 8 9 Crus1 2  - Amygdala | - Parahippocampal G  - Vermis 3 4 5 8 9  - Inferior Temporal G, ant. div. R  - Lingual Gyrus  - Subcallosal Cortex  - Temporal Fusiform C, ant. div. R |
| *Noradrenergic system* | | | | | | | |
| **LC L** | -06 | -32 | -28 | 20600 | 0.000 | - Brainstem  - Lateral Occipital C, sup. div. L  - Frontal Orbital C L  - Middle Temporal G L  - Precuneus C  - Angular G L  - Cerebellum 4 5 6 8 9 Crus1 2  - Inferior Frontal G L  - Temporal Pole L  - Temporal Fusiform C, post. div. L  - Frontal Pole L | - Parahippocampal G, post. div. L  - Putamen L  - Insular C L  - Supramarginal G, post. div. L  - Cingulate G, post. div.  - Vermis 4 5 6 7 8 9 10  - Frontal Operc. C L  - Inferior Temporal G L  - Hippocampus L  - Pallidum L  - Superior Temporal G, ant. div. L |
|  | -24 | +20 | +50 | 3887 | 0.000 | - Frontal Pole L  - Superior Frontal G L | - Middle Frontal GL  - Paracingulate G L |
| **LC R** | +08 | -32 | -34 | 30239 | 0.000 | - Lateral Occipital C R  - Brainstem  - Frontal Pole R  - Precuneus C  - Angular G R  - Cerebellum 3 4 5 6 7b 8 9 10 Crus1 2  - Middle Temporal G R  - Frontal Orbital C R  - Temporal Occipital Fusiform CR  - Lingual G  - Cingulate G, post. div.  - Temporal Pole R  - Supramarginal G, post. div. R | - Occipital Fusiform G R  - Hippocampus R  - Parahippocampal G, post. div. R  - Occipital Fusiform G L  - Superior Parietal Lobule R  - Vermis 4 5 6 7 8 9 10  - Occipital Pole L  - Temporal Fusiform C, post. div. R  - Inferior Temporal G, temporooccipital part R  - Insular C R  - Intracalcarine C |
|  | +42 | +12 | +44 | 2095 | 0.000 | - Middle Frontal G R  - Superior Frontal G R | |
| *^DRN: Dorsalis Raphe Nucleus; k=cluster size; L=left; LC=Locus Coeruleus; NCS= Nucleus Centralis Superior; R=right; SNc=Substantia Nigra pars compacta; VTA= Ventral Tegmental Area; vx=voxels^* | | | | | | | |

**Figure S1.** Seed-based positive bivariate fMRI correlations thresholded at *p*FWE <0.05, corrected for multiple comparisons (not adjusted for the effects of neighboring seeds). **A)** Correlations of individual seeds, **B)** these same correlations (blue) are presented together with semipartial correlation maps (orange). The overlap is presented in purple. In B, numbers indicate the value of the x coordinate.

**A)**

Dorsalis Raphe Nucleus (DRN):


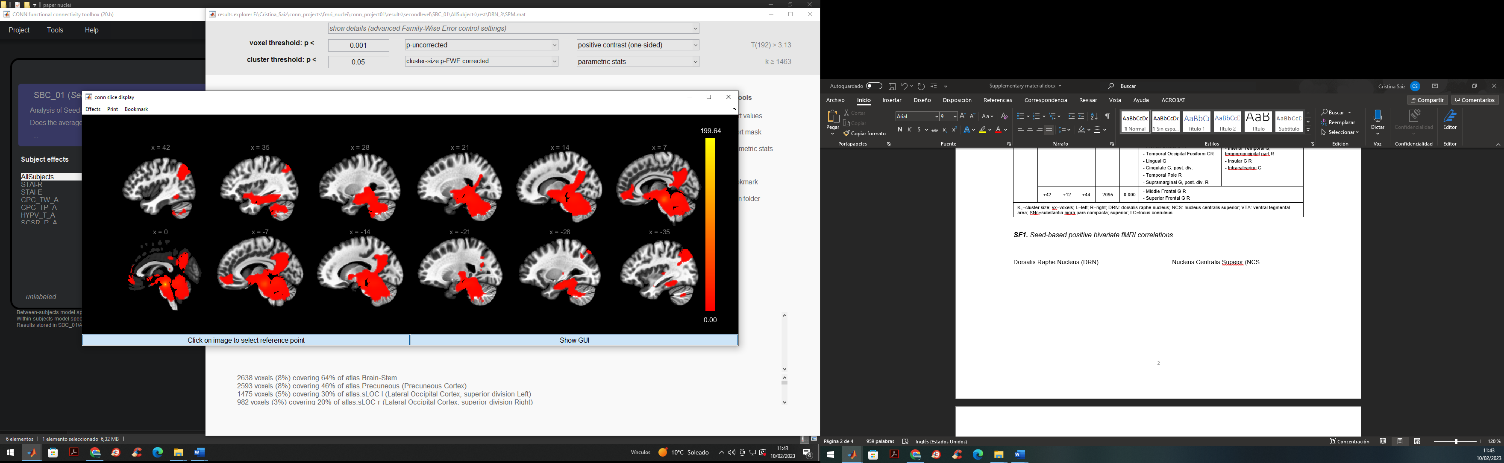


Nucleus Centralis Superior (NCS):


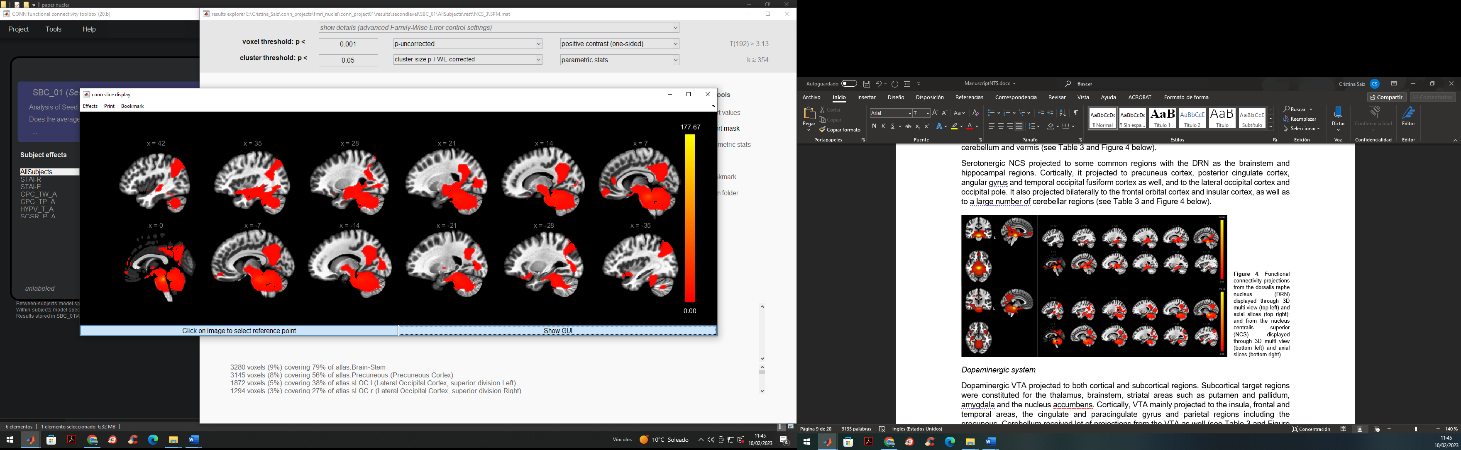


Ventral Tegmental Area (VTA):


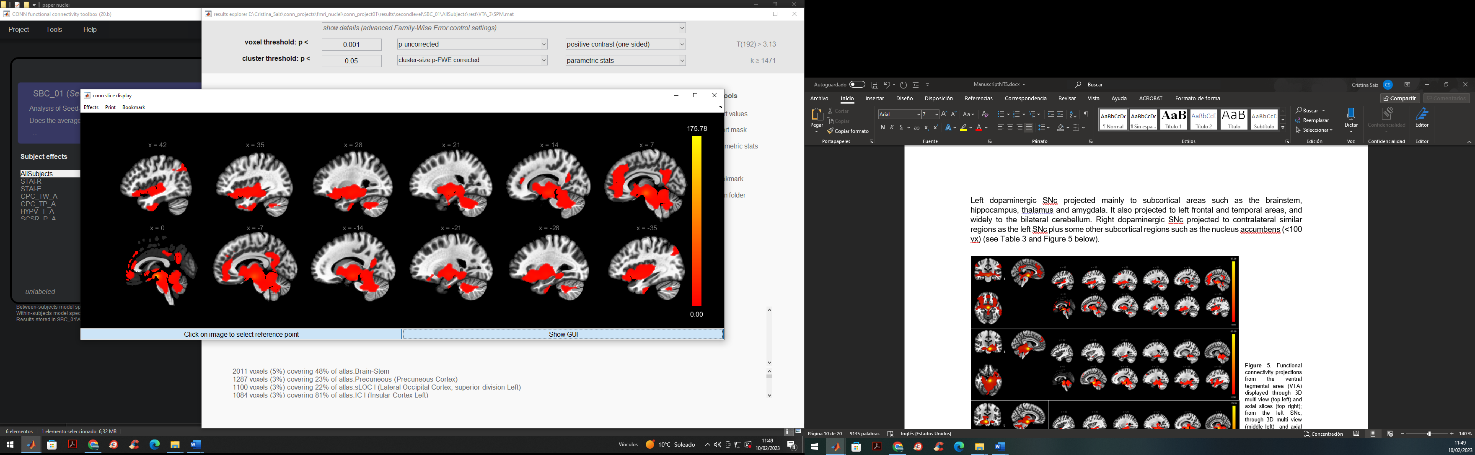


Left Substantia nigra pars compacta (SNc):


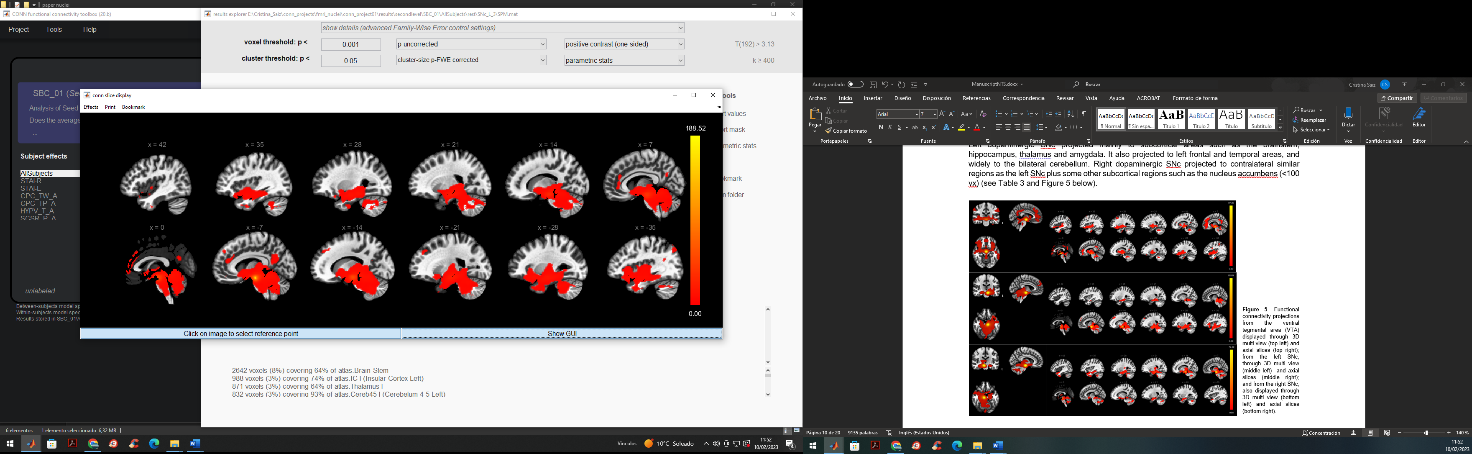


Right Substantia nigra pars compacta (SNc):


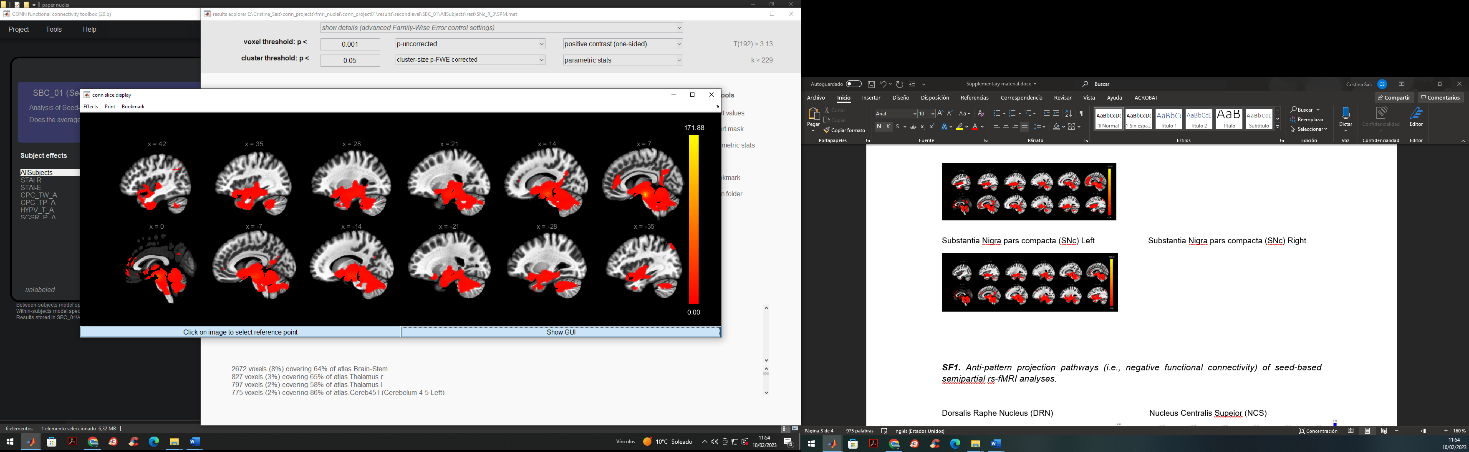


Left Locus coeruleus (LC):


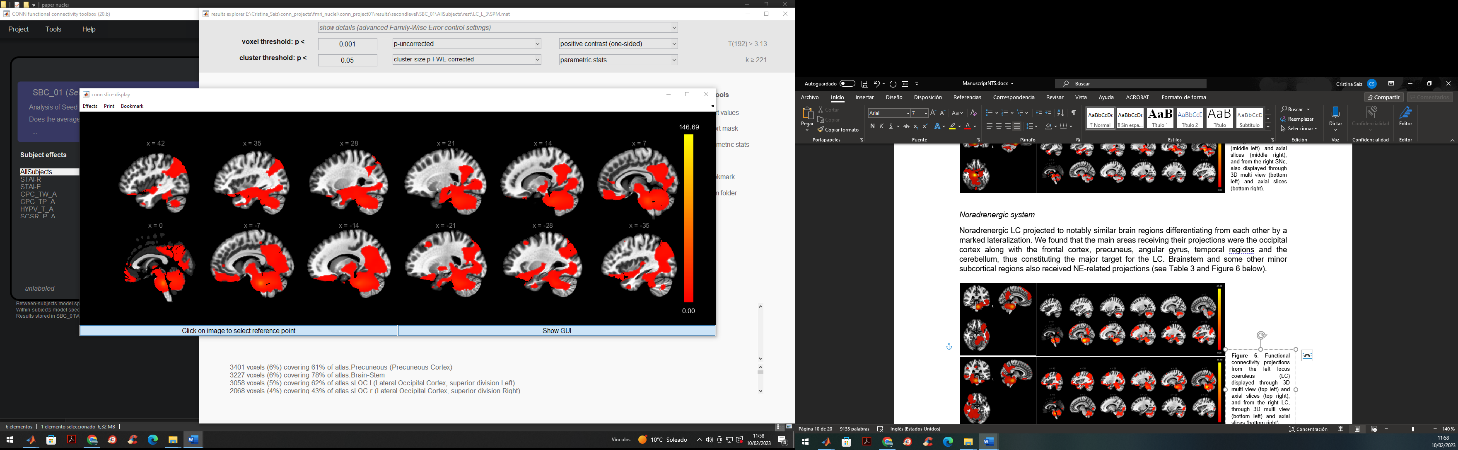


Right Locus coeruleus (LC):


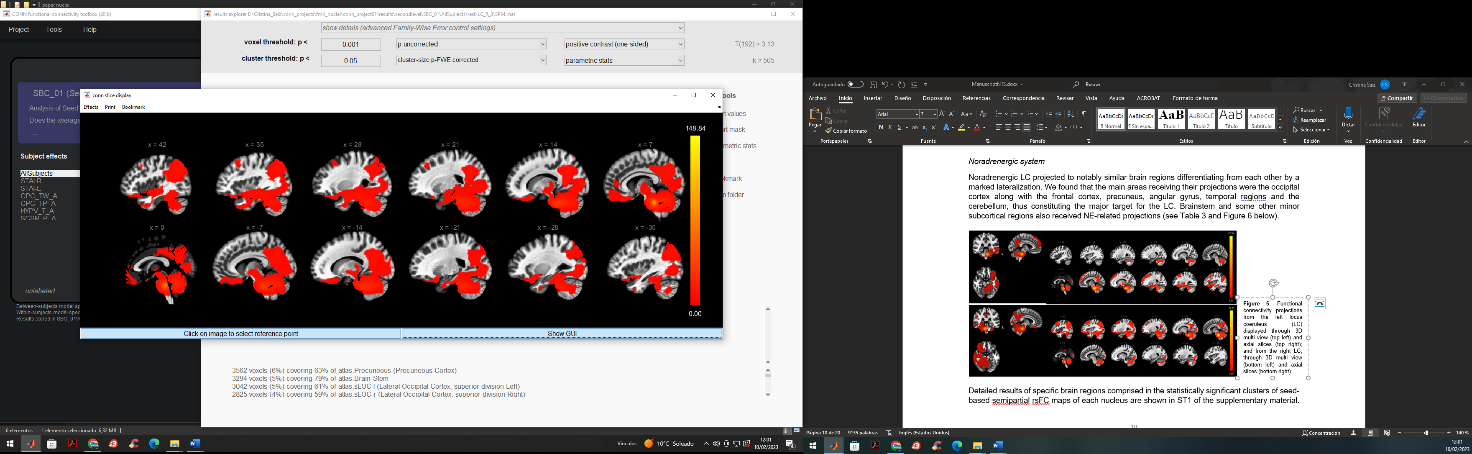


**B)**


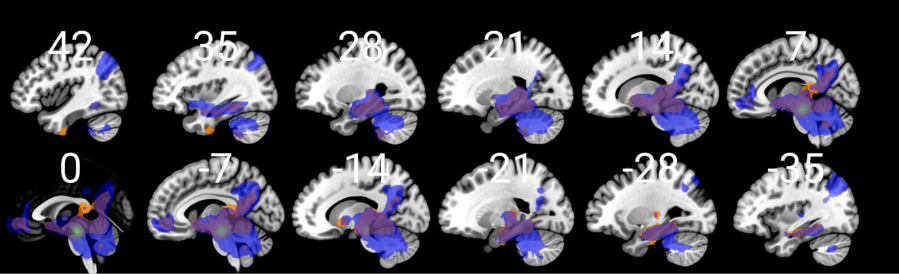
Dorsalis Raphe Nucleus (DRN):

Nucleus Centralis Superior (NCS):


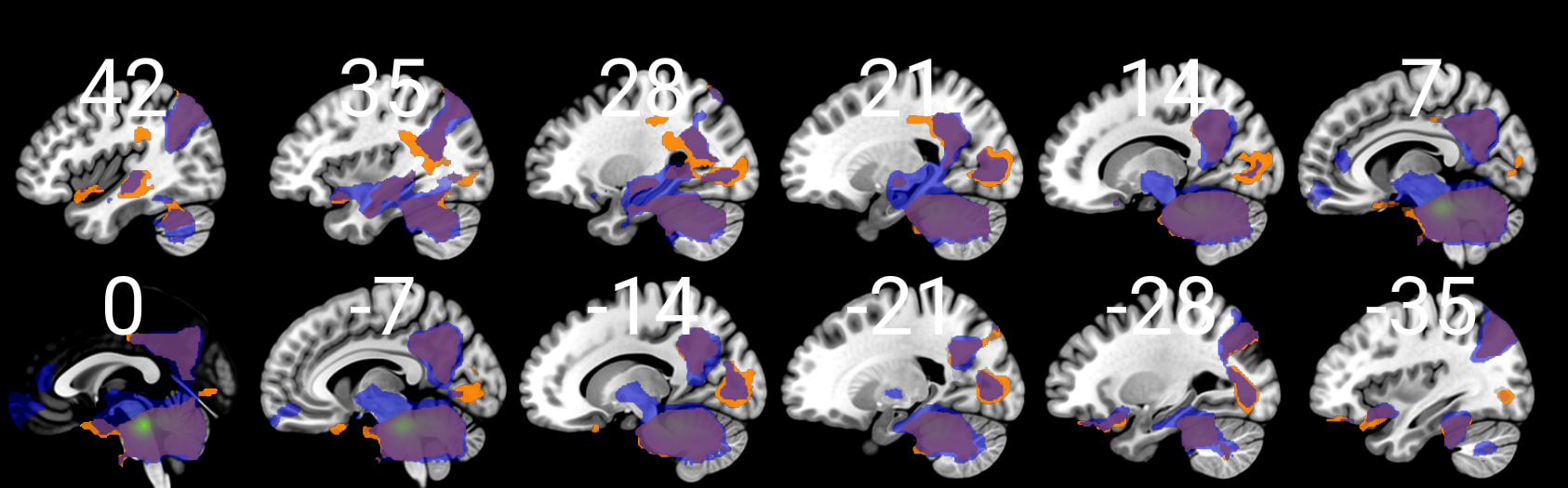


Ventral Tegmental Area (VTA):


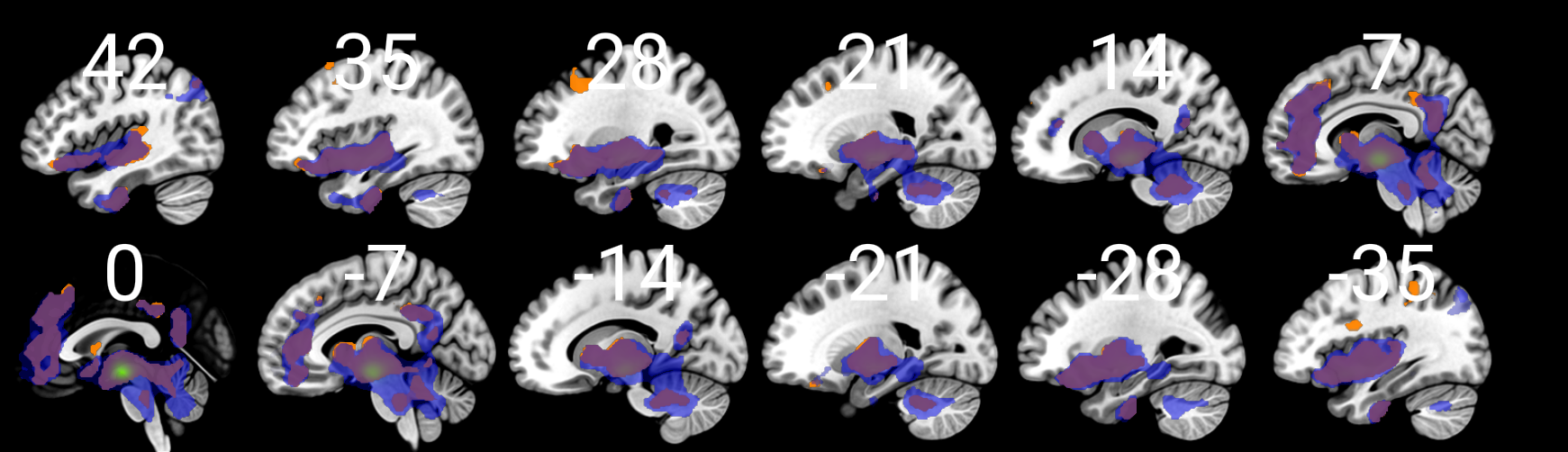


Left Substantia nigra pars compacta (SNc):


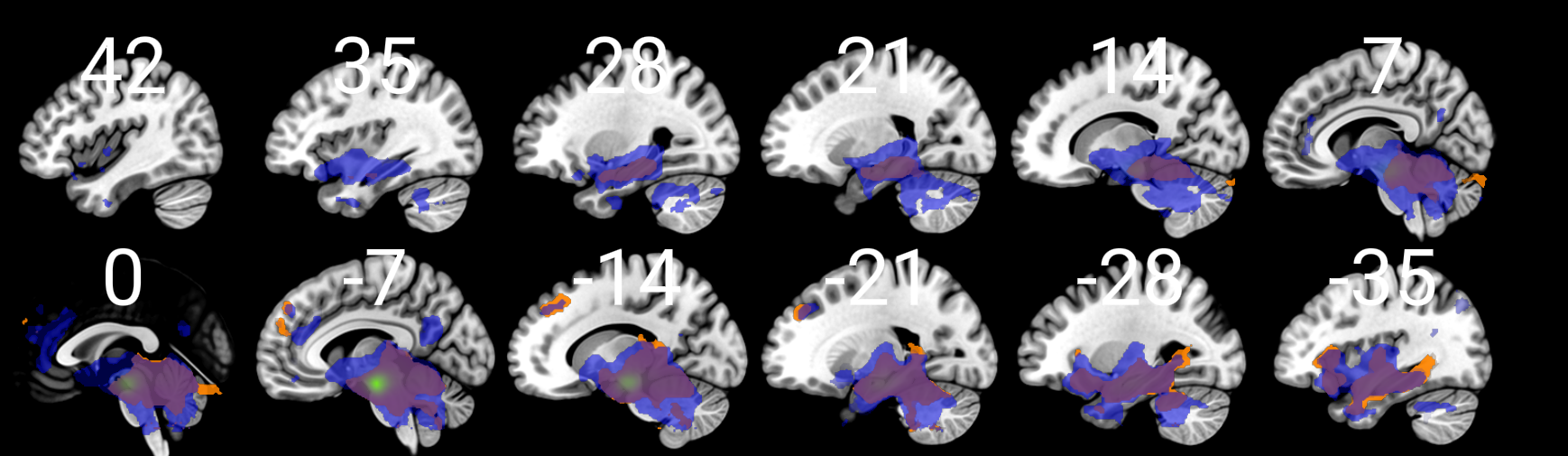


Right Substantia nigra pars compacta (SNc):


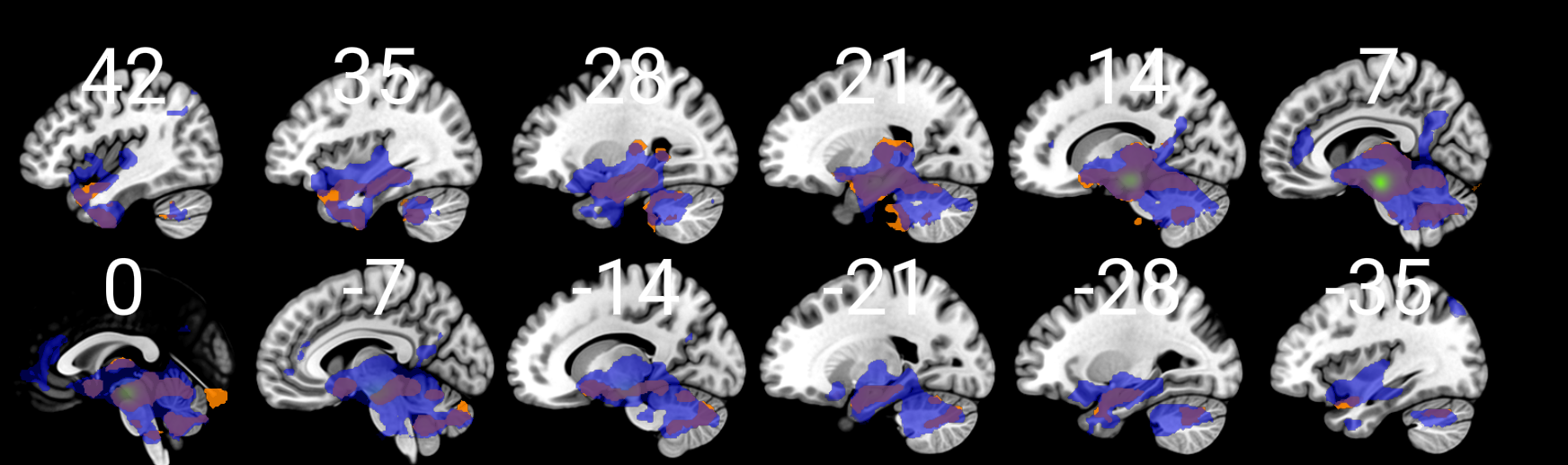


Left Locus coeruleus (LC):


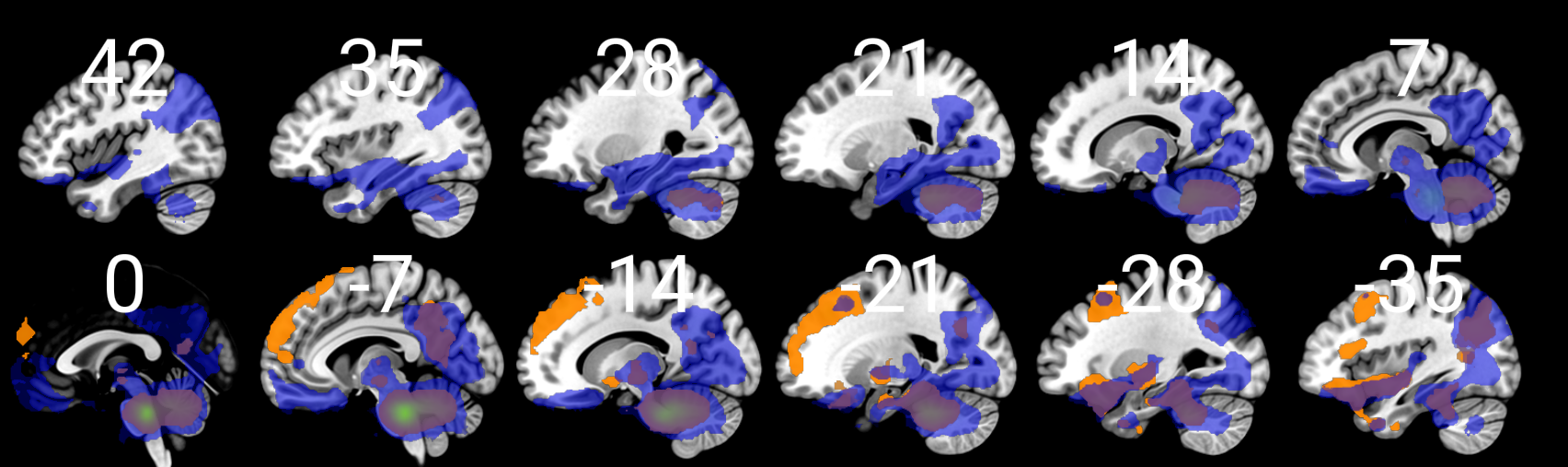


Right Locus coeruleus (LC):


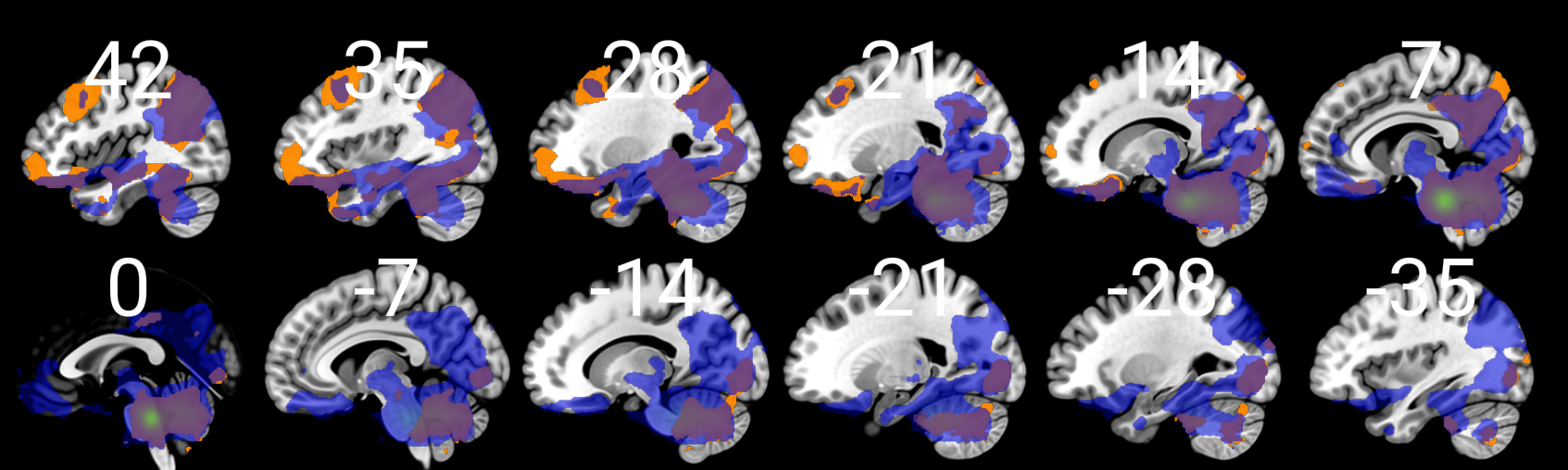


**Figure S2.** Comparison between serotoninergic and dopaminergic FC maps. To enhance visualization and ease comparison between systems, the maps of each serotonergic seed are displayed on the left, alongside the maps of each dopaminergic seed on the right.

1.
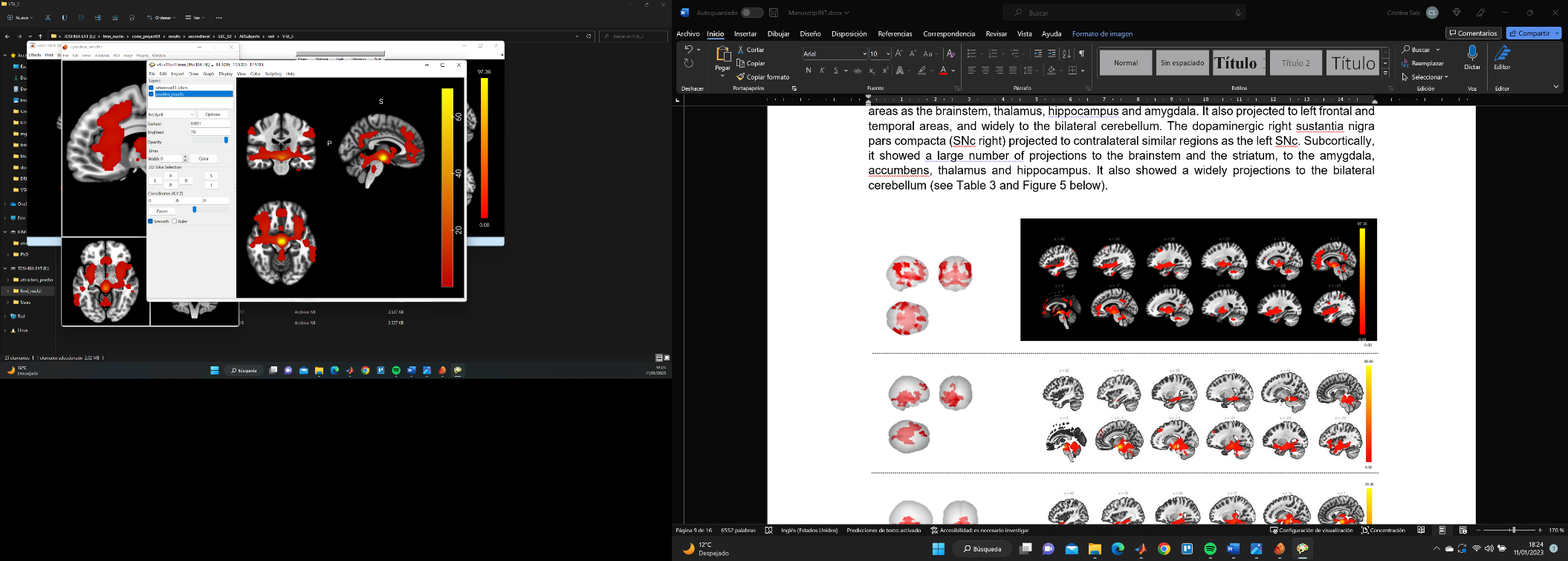
Comparison of the DRN and the VTA FC maps


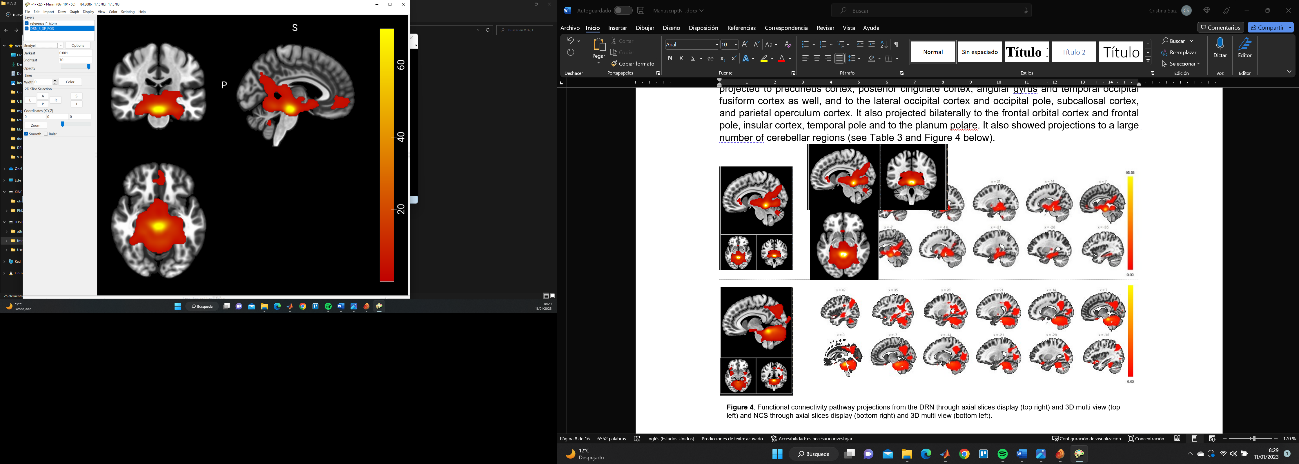


1. Comparison of the DRN and the left SNc FC maps


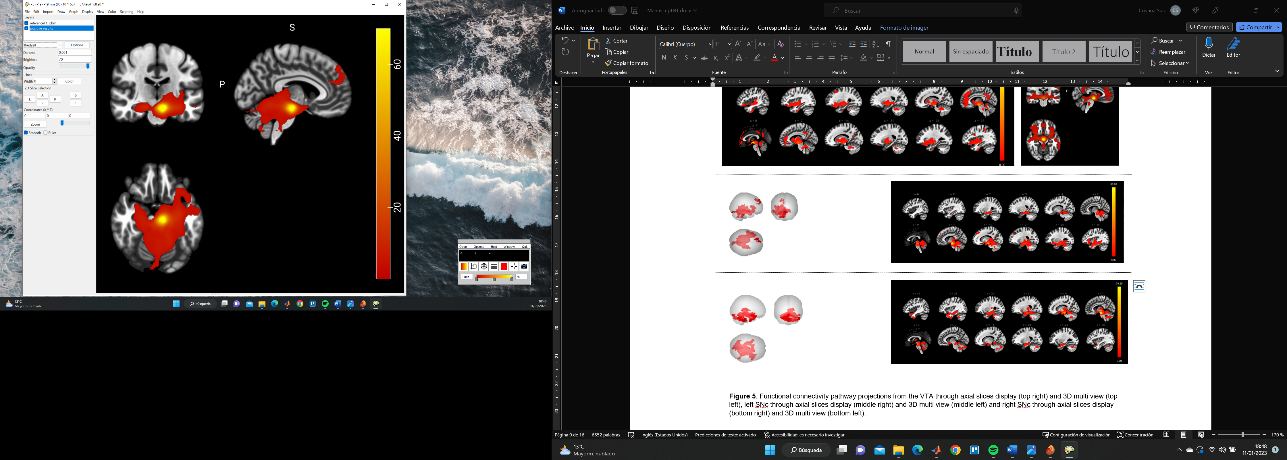

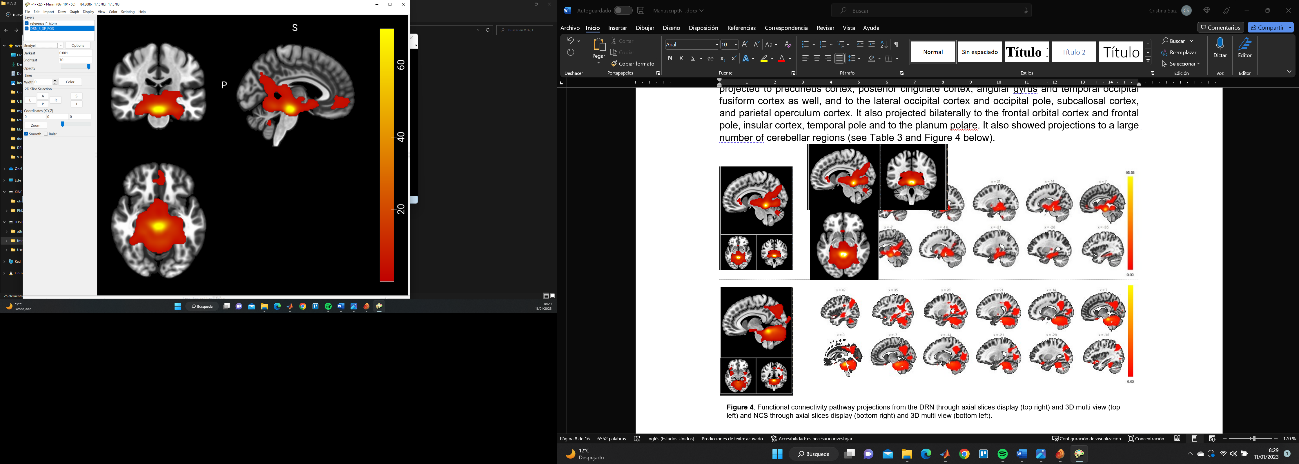


1. Comparison of the DRN and the right SNc FC maps


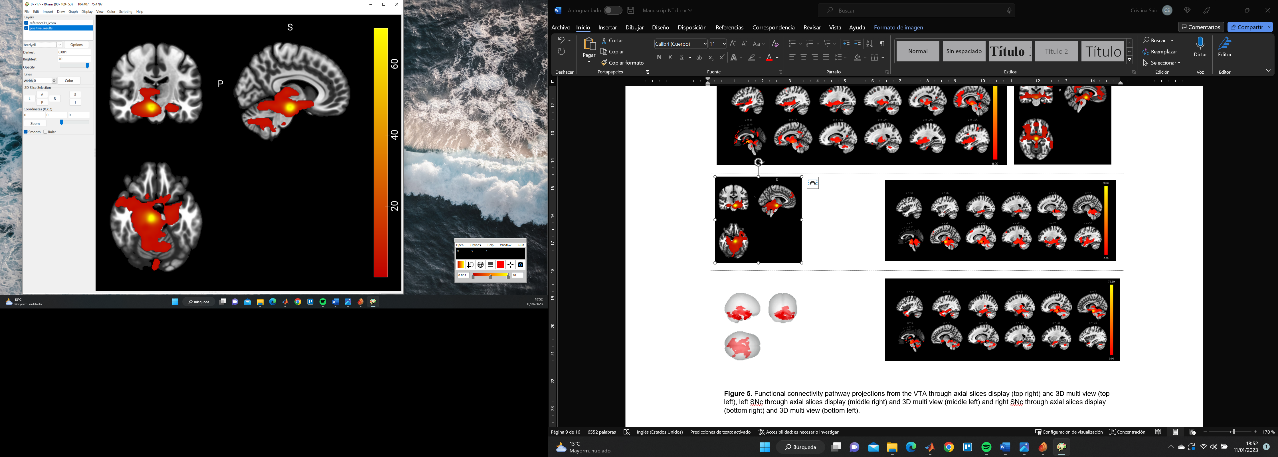

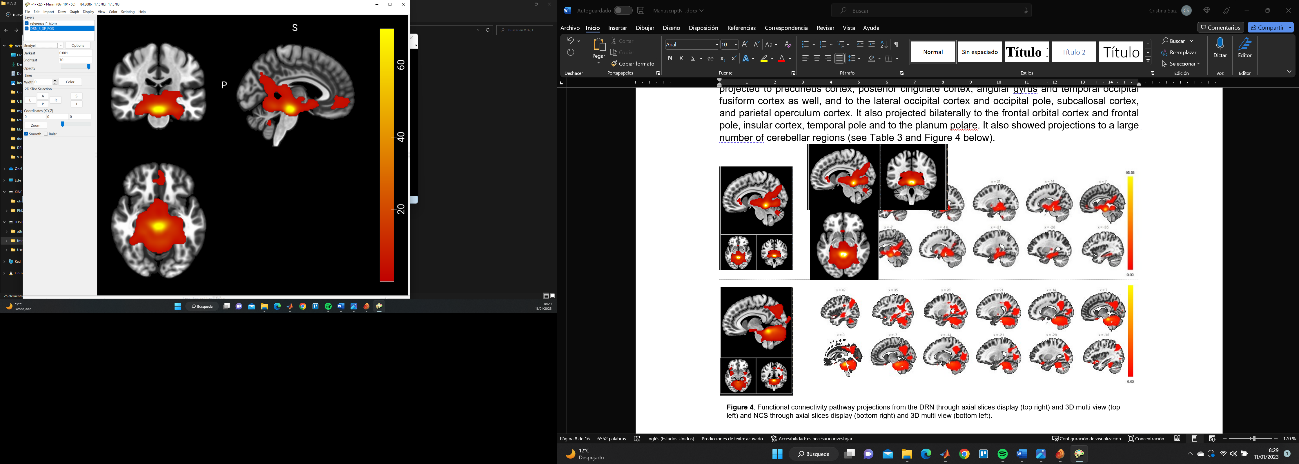


1. Comparison of the NCS and the VTA FC maps


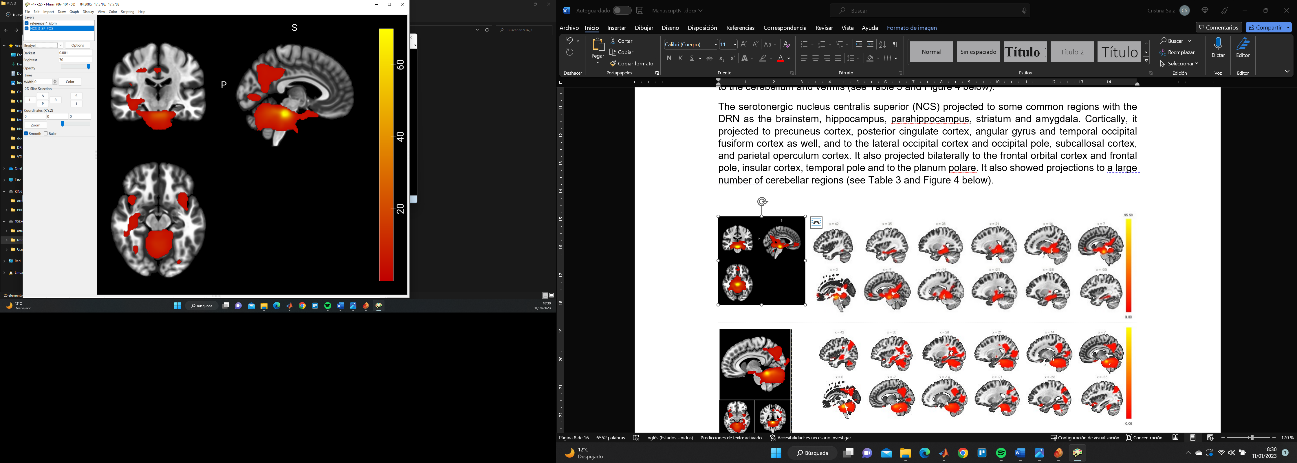

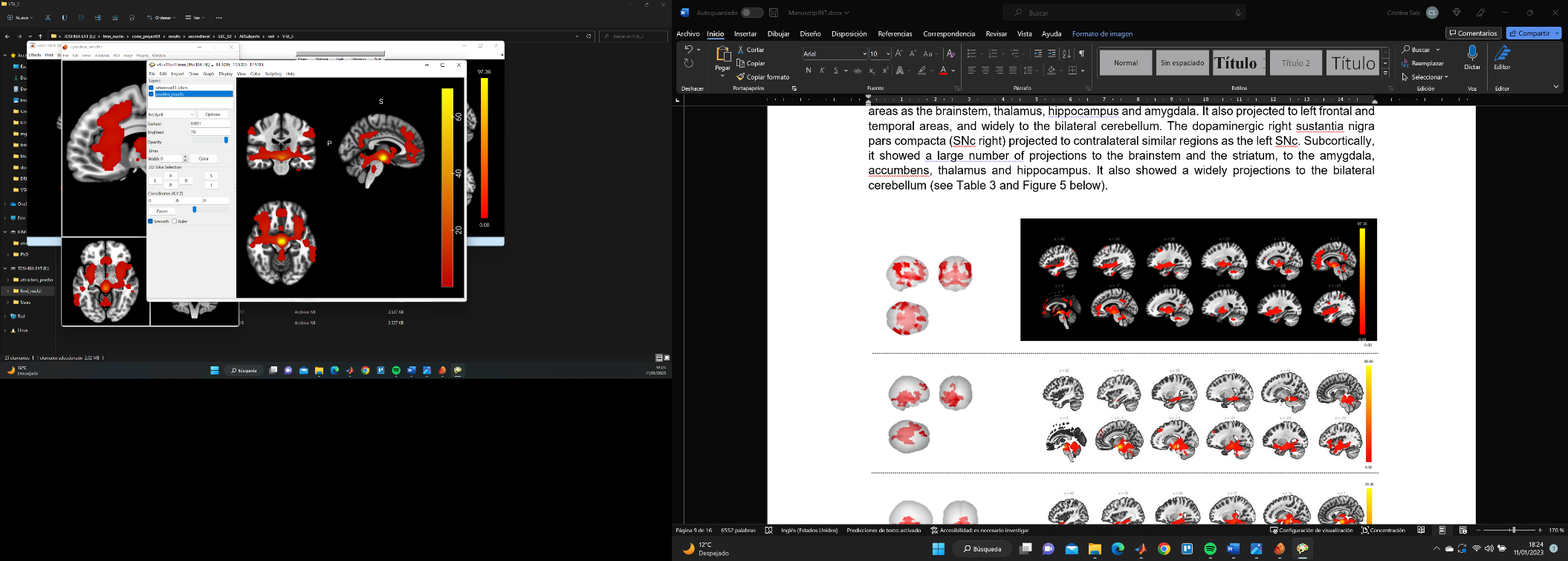


1. Comparison of the NCS and the left SNc FC maps


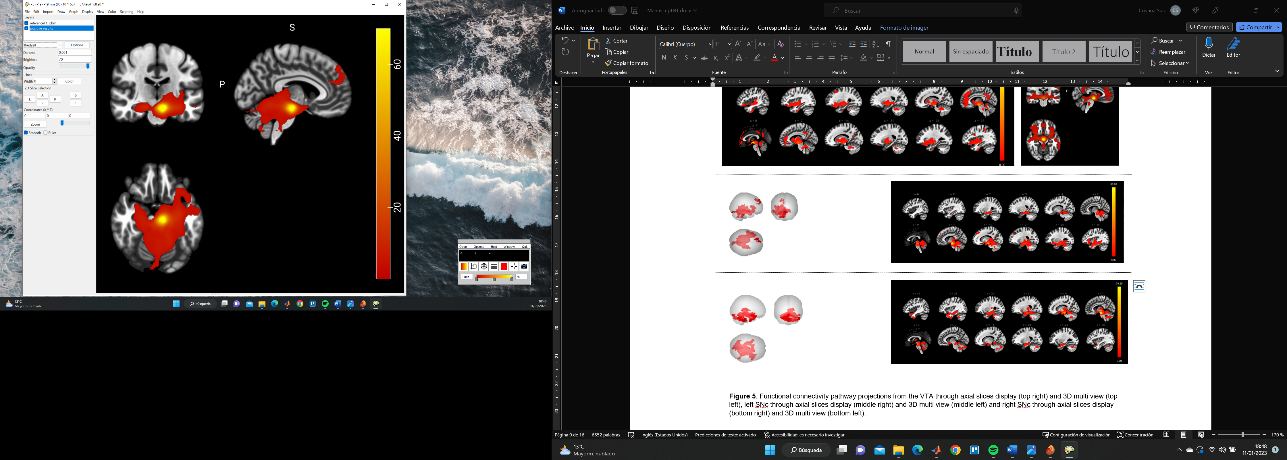

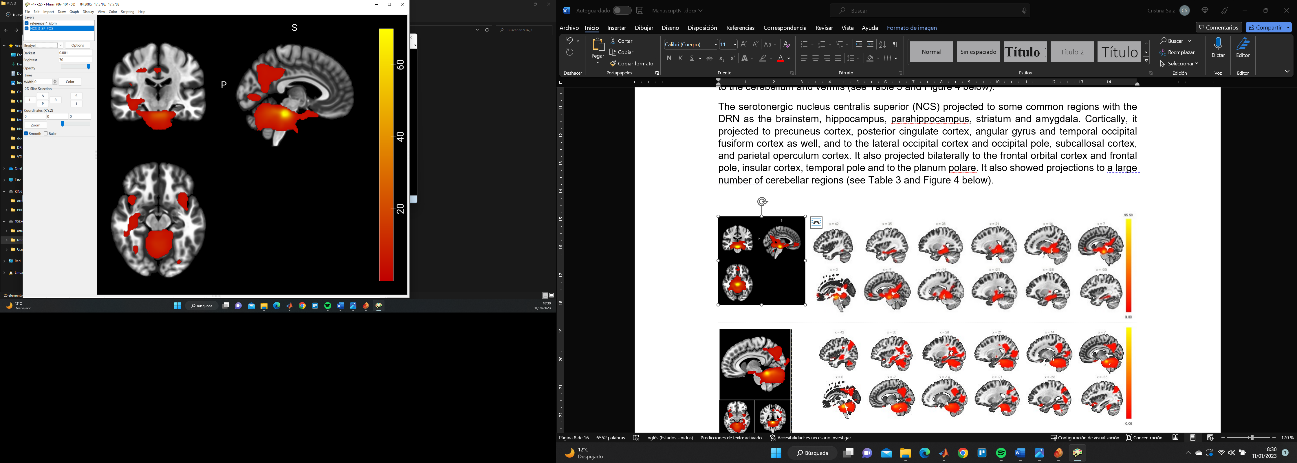


1. Comparison of the NCS and the right SNc FC maps


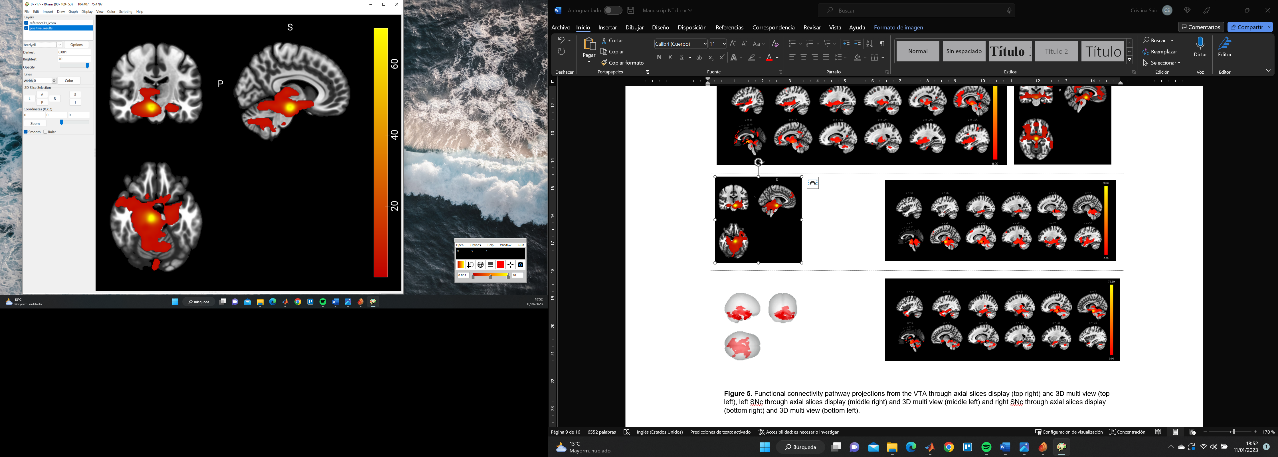

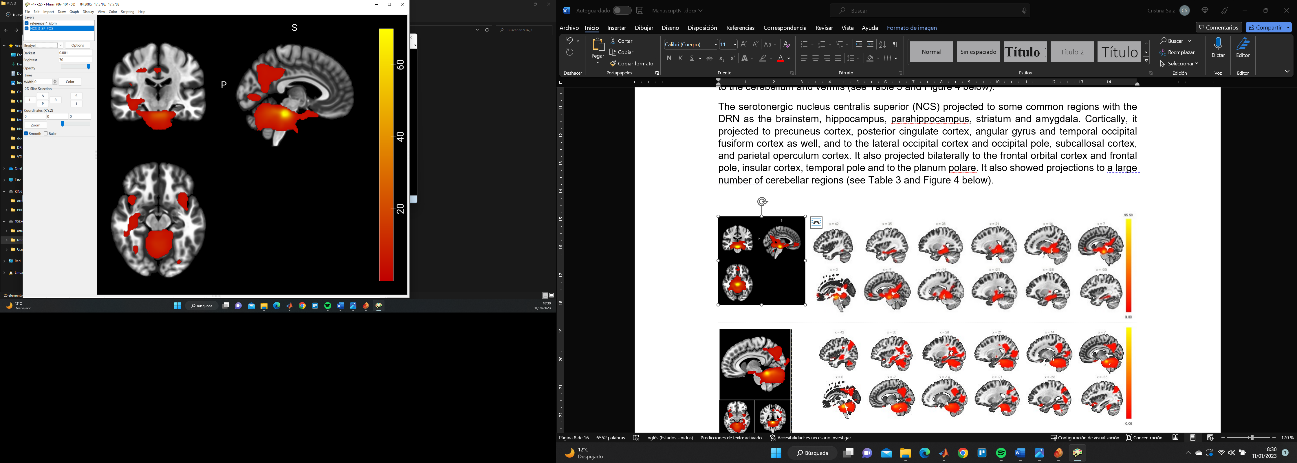


**Figure S3.** Sagittal mosaic view of global connectivity maps: (a) Serotonergic map combining the FC of the DRN and NCS seeds; (b) Dopaminergic map integrating the FC of the VTA, left SNc, and right SNc seeds; (c) Norepinephrine map displaying the joint FC of both LC seeds. Numbers indicate the value of the x coordinate.

a) Global serotonergic system map displaying the DRN and NCS maps together, with the DRN in orange and the NCS in red, utilizing warm color tones.


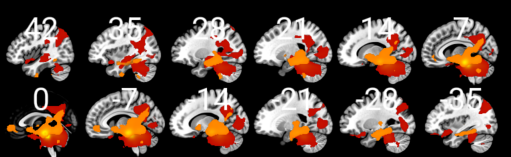


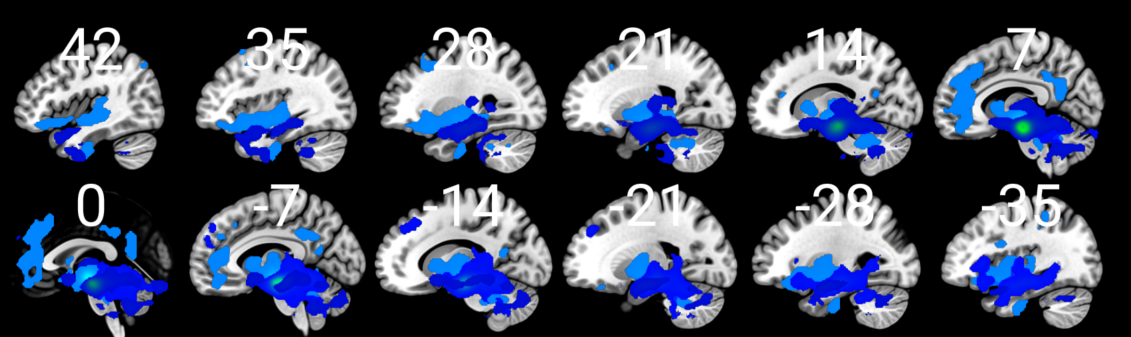
b) Global dopaminergic system map presenting the VTA in light blue, left SNc in medium blue, and right SNc in medium-dark blue, depicted together in a palette of cool colors.

c) Global noradrenergic system map displaying the left LC in dark purple and the right LC in bluish purple, represented together in shades of purple.


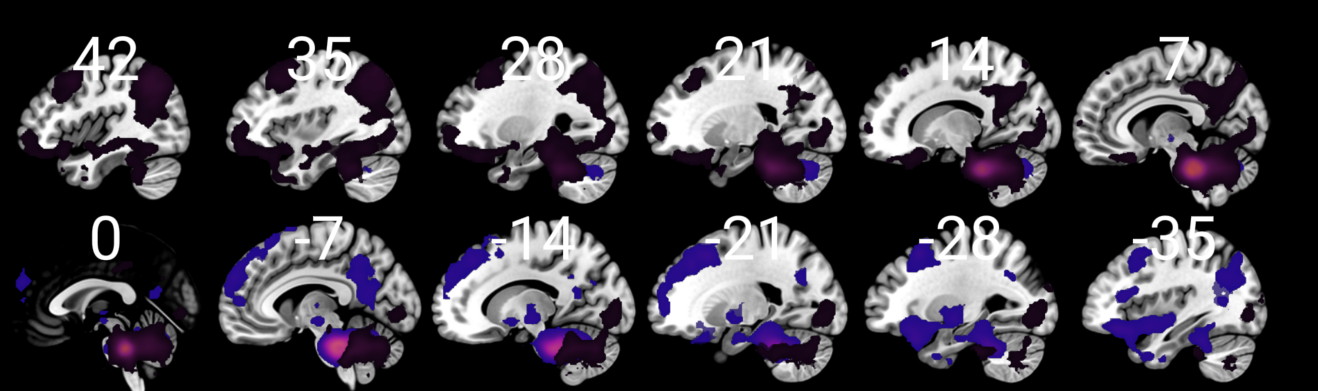


**Figure S4.** Plots displaying all significant and non-significant correlations, both positive and negative, from all Cross-modal correlations between rsFC and nuclear imaging neurotransmitter maps.

Serotonergic system

Dorsalis Raphe Nucleus (DRN): Nucleus Centralis Superior (NCS):


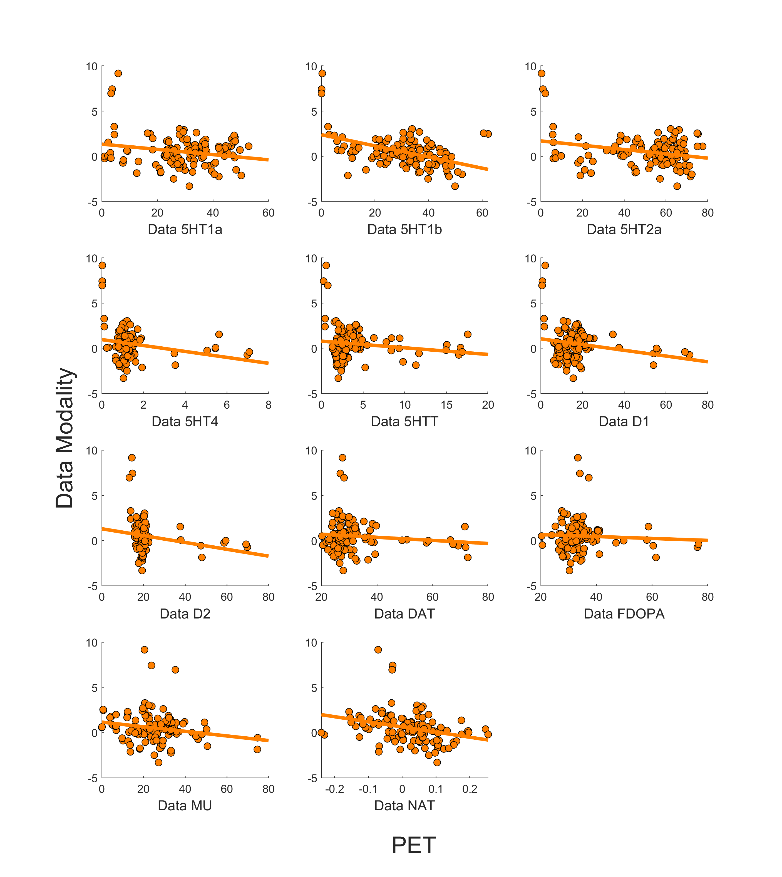

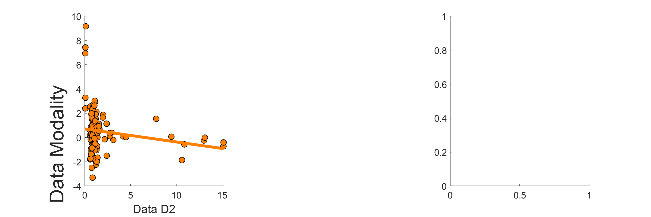

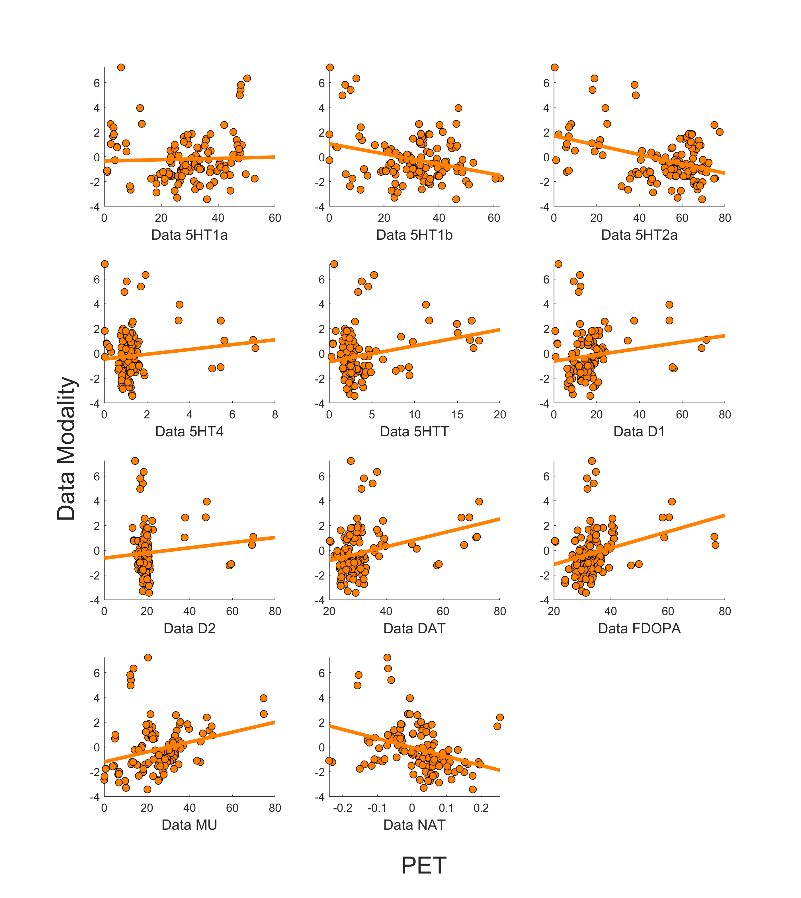

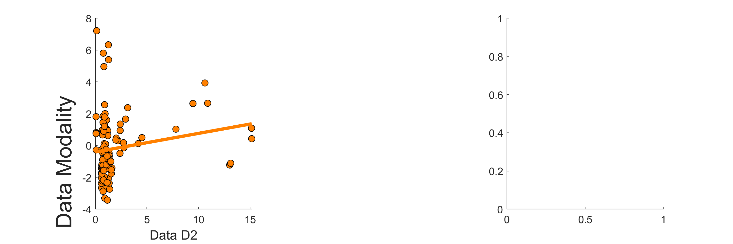


Dopaminergic system

Ventral Tegmental Area (VTA): Left Substantia nigra pars compacta (SNc):


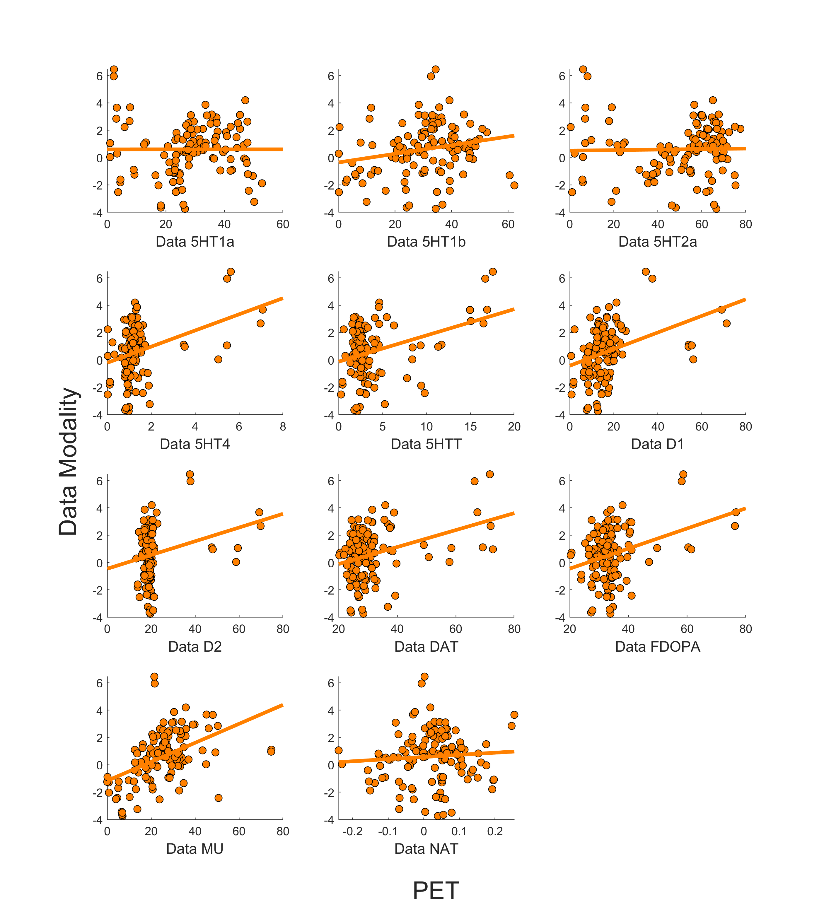

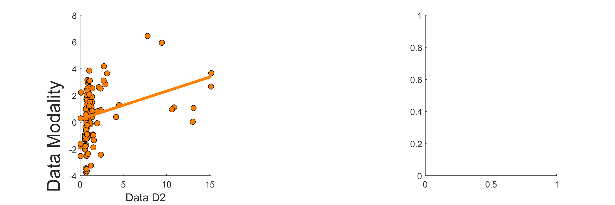

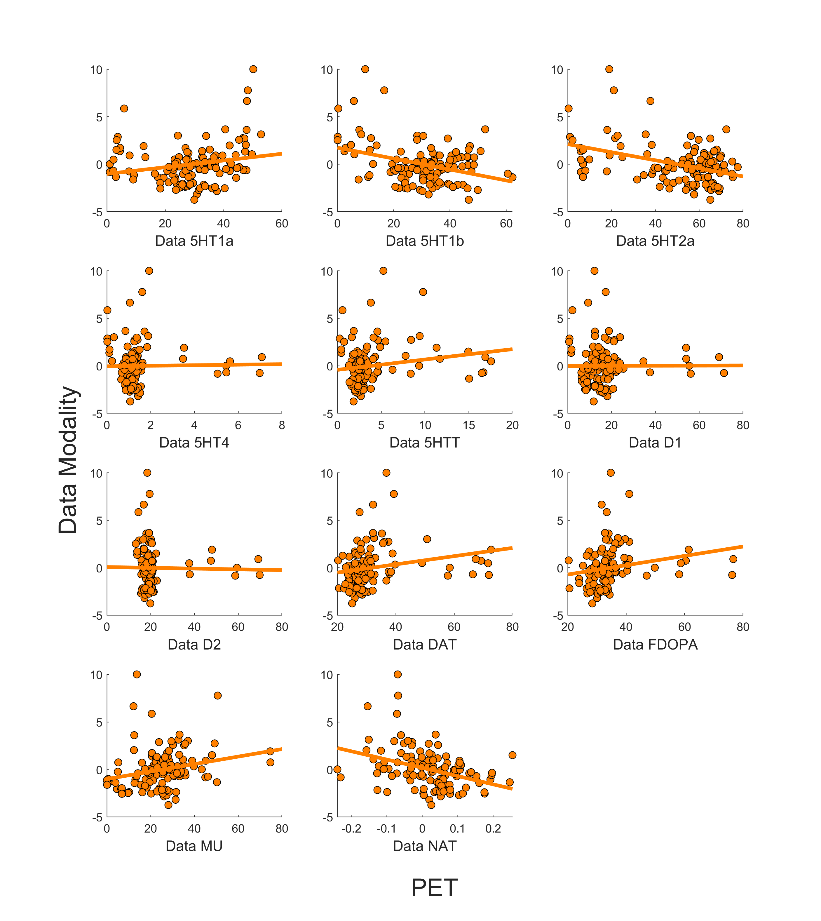

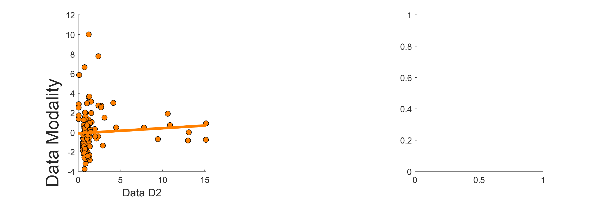


Right Substantia nigra pars compacta (SNc):


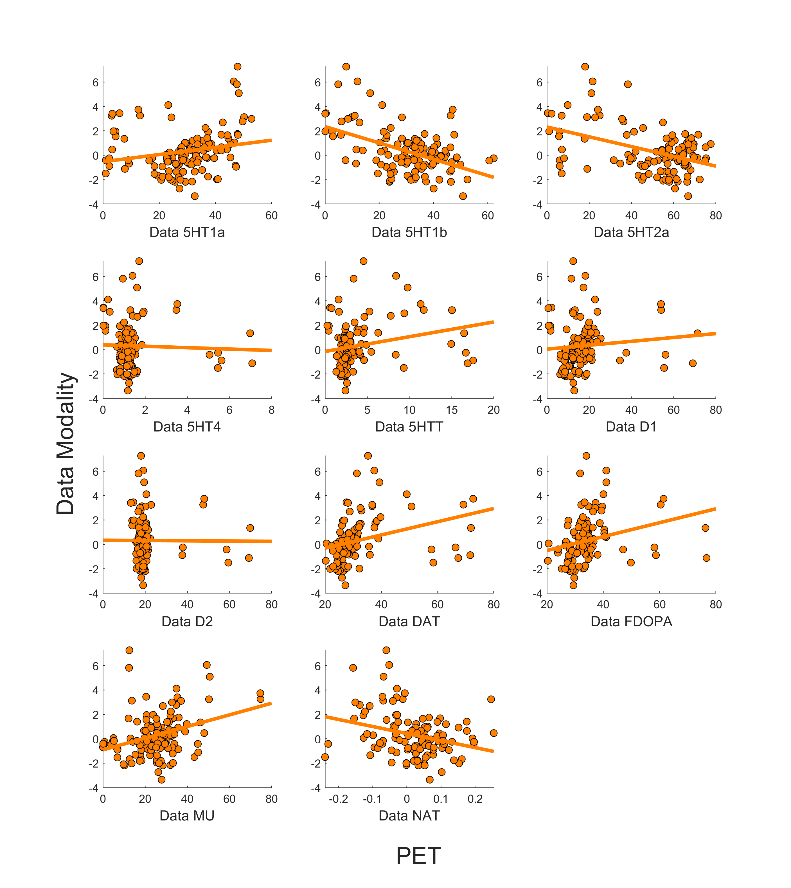

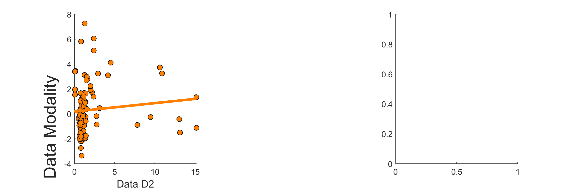


Noradrenergic system

Left Locus coeruleus (LC): Right Locus coeruleus (LC):


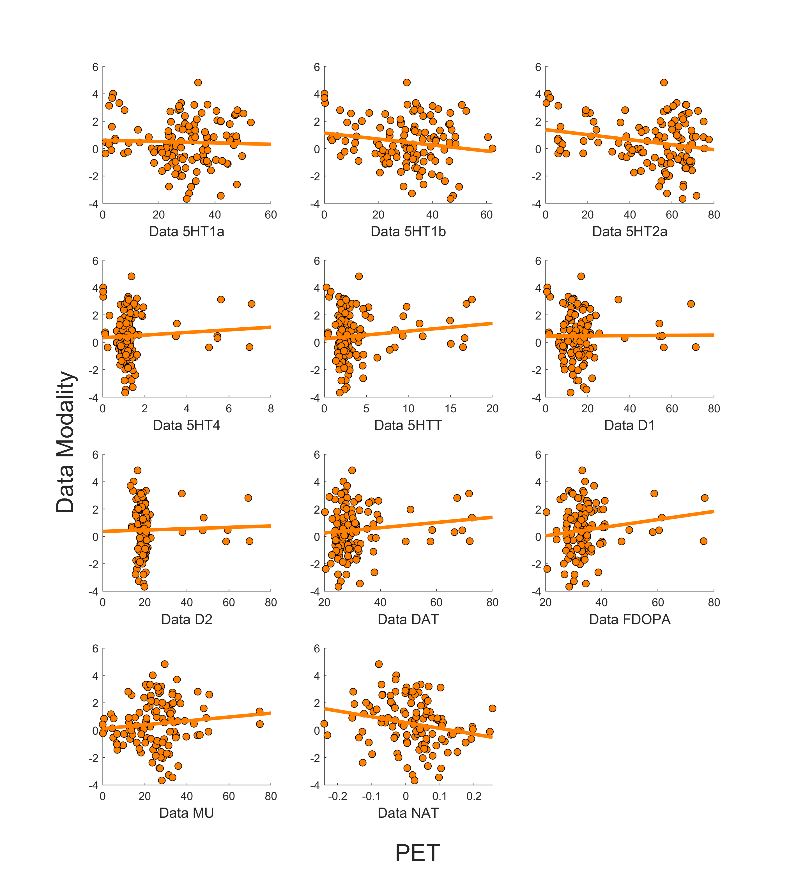

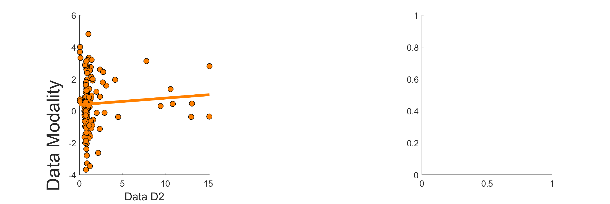

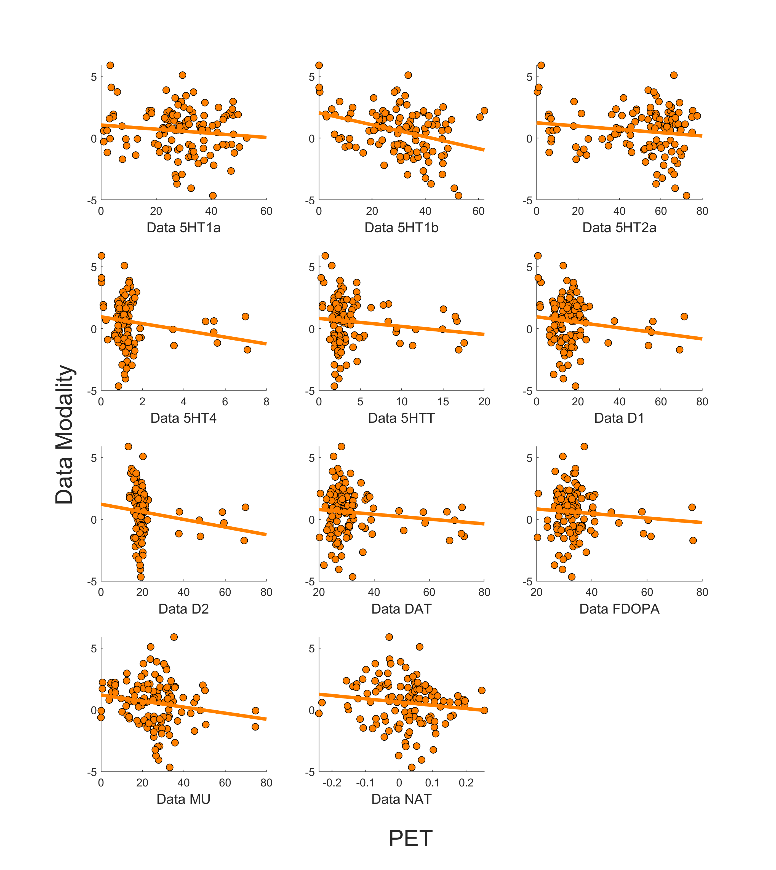

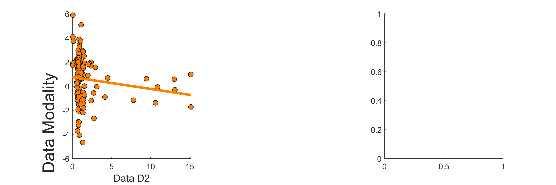


**Table S2**. Significant positive spatial correlations between rsFC maps and nuclear imaging maps of neurotransmitter-related compounds, corresponding to the findings reported in Figure 5. Statistical significance corresponds to Pearson’s correlation value. Non-significant findings according to Spearman’s correlation value, are highlighted in red.

| **Table S2**. Significant positive correlations between rsFC maps and the nuclear imaging maps | | | | |
| --- | --- | --- | --- | --- |
| Nuclear imaging map | Neurotransmitter system-related | Statistics | | |
|  |  | Pearson r | *FDR p*-value | Spearman rho |
| *DRN FC map* | | | | |
| 5HTT | 5-HT | 0.271 | 0.011 | 0.003 |
| DAT | DA | 0.304 | <0.001 | 0.167 |
| FDOPA | DA | 0.270 | 0.011 | 0.221 |
| *VTA FC map* | | | | |
| 5HT_4_ | 5-HT | 0.357 | <0.001 | 0.329 |
| 5HTT | 5-HT | 0.345 | <0.001 | 0.074 |
| D_1_ | DA | 0.412 | <0.001 | 0.445 |
| D_2_ | DA | 0.329 | <0.001 | 0.447 |
| DAT | DA | 0.373 | <0.001 | 0.247 |
| FDOPA | DA | 0.373 | <0.001 | 0.173 |
| MU | DA | 0.582 | <0.001 | 0.525 |
| *Left SNc FC map* | | | | |
| 5HTT | 5-HT | 0.246 | 0.021 | 0.126 |
| *Right SNc FC map* | | | | |
| 5HTT | 5-HT | 0.330 | <0.001 | 0.187 |
| DAT | DA | 0.306 | <0.001 | 0.364 |
| *^5-HT: serotonin; 5-HTT: serotonin transporter; D= dopamine; DAT: dopamine transporter; DRN= dorsal raphe nucleus; FC= functional connectivity; FDOPA: fluorodopa; MU: Mu opioid receptor; SNc = substantia nigra pars compacta; VTA = ventral tegmental area.^* | | | | |
